# Chunking as a rational solution to the speed–accuracy trade-off in a serial reaction time task

**DOI:** 10.1038/s41598-023-31500-3

**Published:** 2023-05-11

**Authors:** Shuchen Wu, Noémi Éltető, Ishita Dasgupta, Eric Schulz

**Affiliations:** 1 MPRG Computational Principles of Intelligence, Max Planck Institute for Biological Cybernetics, Tübingen, Germany; 2 Department of Computational Neuroscience, Max Planck Institute for Biological Cybernetics, Tübingen, Germany; 3 Google DeepMind, New York City, NY USA

**Keywords:** Cognitive neuroscience, Computational neuroscience, Learning and memory, Psychology, Human behaviour

## Abstract

When exposed to perceptual and motor sequences, people are able to gradually identify patterns within and form a compact internal description of the sequence. One proposal of how sequences can be compressed is people’s ability to form chunks. We study people’s chunking behavior in a serial reaction time task. We relate chunk representation with sequence statistics and task demands, and propose a rational model of chunking that rearranges and concatenates its representation to jointly optimize for accuracy and speed. Our model predicts that participants should chunk more if chunks are indeed part of the generative model underlying a task and should, on average, learn longer chunks when optimizing for speed than optimizing for accuracy. We test these predictions in two experiments. In the first experiment, participants learn sequences with underlying chunks. In the second experiment, participants were instructed to act either as fast or as accurately as possible. The results of both experiments confirmed our model’s predictions. Taken together, these results shed new light on the benefits of chunking and pave the way for future studies on step-wise representation learning in structured domains.

## Introduction

William James famously said that we are born into a “blooming, buzzing confusion”, and that we escape that confusion by gradually making sense of the series of events we perceive. How we perceive a sequence of perceptual stimuli, process them, and extract underlying structure, is a fundamental question of psychological investigations. One proposal of how the blooming, buzzing confusion of seemingly disparate sequential events can become one cognitive unit is chunking^1–4^. Upon exposure to sequential stimuli, humans and animals can identify repeated patterns and segment sequences into chunks of patterns^5^. To this end, separate sequential elements merge into one cognitive entity. This cognitive entity is then recalled and identified as a whole^6^: a phenomenon known as *chunking*^7,8^.

Chunking is a phenomenon spanning across sequence learning, grammar learning, visual and working memory tasks, and function learning, among others^8–12^. The ability to discover statistical regularities in sequences, and to identify them as discrete, disparate units of chunks enables us to form a compact and compressed memory representation^13^, readily transferable to novel domains^14^, and enables us to progress from novices to experts^15,16^. As primitive building blocks of cognitive construction units, a complex and lengthy sequence reduces to several chunks. This property facilitates the organization of actions^7^, and can subsequently help with compositionality in learning^17^, communication of structure^18^, hierarchical planning^19^ and others. In short, chunking is a critical and universal learning phenomenon. Here we propose another benefit of chunking in sequential tasks: the ability to more easily predict future outcomes and thereby act faster. Thus, our work connects the literature of chunking with that of the speed-accuracy trade-off.

**Figure 1:**
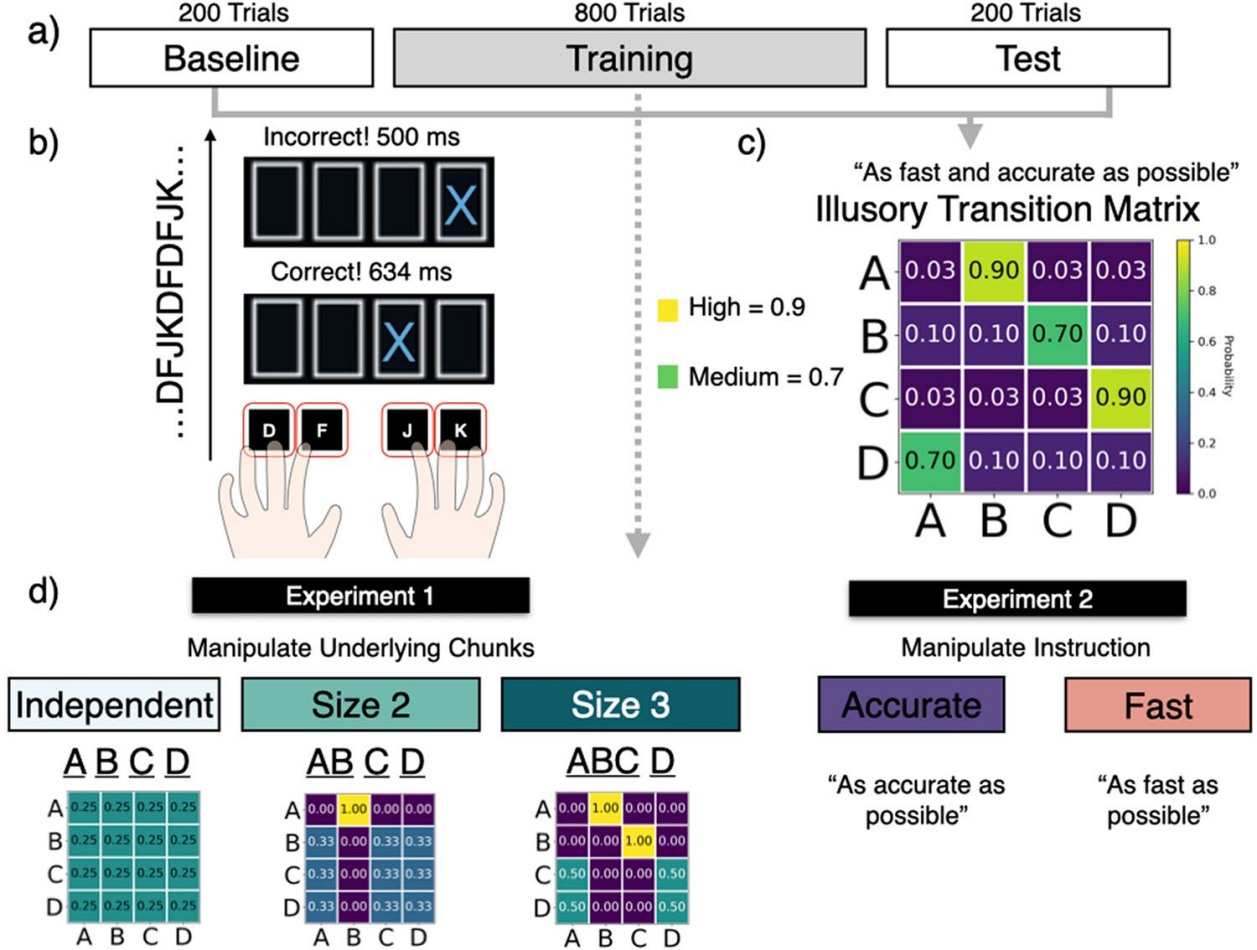
(**a**) Task structure for both experiments. Six training blocks are sandwiched between two baseline and two test blocks. The baseline and test blocks contain sequences generated from the “illusory” transition matrix in (**c**). (**b**) Participants are instructed to press the corresponding key on the keyboard according to trial-by-trial displayed instructions. They are given feedback on their performance, including accuracy and reaction times before the subsequent trial. (**c**) A non-deterministic, “illusory” transition matrix of the four possible key-presses is used to generate sequences for the baseline and test blocks for both experiments. The generative transition matrix with the two high (from A to B, C to D) and two medium transition probabilities (from B to C, D to A) produces “illusory” chunks that can be perceived as frequently occurring. To control the effect of habitual presses from consecutive fingers, a random mapping from “A”, “B”, “C”, “D” to “D”, “F”, “J”, “K”, is generated independently for each participant. (**d**) The instructions for training blocks differed between the two experiments and corresponding groups. In Experiment 1, participants were divided into three groups who learned independent, size 2, and size 3 chunks from a predefined set of chunks with equal probability. In Experiment 2, the sequences in the training blocks were also generated from the “illusory” transition matrix. One group was instructed to act as accurately as possible and the other groups was instructed to act as fast as possible.

The speed-accuracy trade-off is observed both in humans and animals across various task domains^20^. When speed is emphasized, participants in both lab and naturalistic settings tend to make more mistakes while reacting faster than when accuracy is emphasized^21,22^. While earlier work focused on analyzing reaction times and accuracy^21,23^, little work has been done to relate the speed-accuracy trade-off to chunking and examine it affects the process and outcome of learning representations.

The serial reaction time task (SRT), a classical paradigm to study motor sequence learning^12,24–26^, is ideal for studying the speed-accuracy trade-off and chunking. In SRTs, sequences of instruction cues appear consecutively on the screen, after which participants react by pressing the corresponding key that maps to the cue. If particular patterns, for example, ABC, keep repeating, then grouping repeated chunks as a unit facilitates the prediction of upcoming sequences. The detection of a chunk’s beginning, in this case, A, implies that the within-chunk items B and then C will follow. This anticipation of the following elements of a given chunk can allow participants to anticipate what is coming next and thereby react faster^12,14^. Chunking sequence elements, however, can also come at a cost when the sequence is probabilistic. By assuming deterministic transitions between the within-chunk items AB, participants might lose fine-grained statistical information about single-item instructions and thereby occasionally miss between-chunk transitions such as AC. This, in turn, can decrease their accuracy.

We propose a model that trades off between speed and accuracy when performing SRTs. Our model calculates the utility of acquired chunk representations as a weighted sum of how well they capture the statistical structure in the SRT (accuracy) and whether they permit faster responses (speed). Our model then iteratively decides whether or not to chunk consecutive items. This model makes two distinct predictions. First, in environments where deterministic chunks exist, adding them to the representation is beneficial because they speed up reaction times without losing accuracy. Thus, people should chunk more in environments with more or longer chunks. In our first experiment, we tested this prediction by training participants on sequences containing underlying chunks. We designed a couple of analysis methods to test and verified this prediction. The results of this experiment suggest that participants adapt their chunking behavior to the underlying chunks in the sequence when they are given universal instructions to act as fast and accurately as possible. A subsequent prediction following the first experiment from the model is that when participants are given distinct instructions to perform on the task, these instructions will induce distinct chunking behavior even when the sequence have the same underlying statistics for the two groups. Specifically, this will be a rational strategy for the model to learn chunks in cases where the underlying environment is non-deterministic and does not contain any chunks. As the utility of speed increases (at the cost of accuracy), participants might also chunk consecutive elements more often and learn longer chunks. Since chunking frequently co-occurring events improves reaction time at the cost of overall accuracy, chunking can be a rational strategy to act faster. We tested and verified this prediction in a second experiment by training participants on sequences generated from a first-order Markovian transition matrix with “illusory” chunks while instructing one group to focus on speed and the other group to focus on accuracy. The results of this second experiment suggest that the group focusing on speed chunked more than the group focusing on accuracy. The fast group learns more chunks and makes more mistakes. While the accurate group learns the underlying generative model of the sequence better, but smaller chunks than the fast group. Our results shed new light on the benefits of chunking under specific task instructions and pave the way for future studies on structural inference in statistical learning domains.

### Serial reaction time task

We study chunking in a serial reaction time task (SRT, see Fig. 1b). Participants are instructed to press keys corresponding to a sequence of cues that appear on the screen. The instruction cross turns green after a correct keypress and red after an incorrect keypress. The subsequent trial starts after a 500ms response-to-stimulus interval. The task starts with two baseline blocks followed by six training blocks and ends with two test blocks. Each block consists of 100 trials. For both experiments, the same generative mechanism produces the baseline and the test blocks. To study whether participants’ chunking behavior adapts to task demands in an SRT task, we manipulate various properties of the training blocks to examine how they affect behavior in the test block, using the baseline block as a comparison. The observed differences between the test and baseline blocks reflect the changes in representations elicited by the training blocks.

There are various approaches to generating sequences in an SRT paradigm. One type of instruction involves repeated sequence^26,27^, while others avoid direct repetitions or runs such as 1234^28^, where 1,2,3,4 refer to 4 targets on the computer screen. One probabilistic way of generating the sequence is the alternating serial reaction time task^29,30^, where instruction patterns can be 1r4r3r2r, with r being a randomly chosen target. Other probabilistic ways of generating the presented sequences include choosing successive images according to a probabilistic first-order Markov transition process, specified by a conditional probability matrix^31^. Schvaneveldt and Gomez used two sequences, such as 1243 and 1342 and drew the target sequence via weighted coin flip results^32^. Several reasons have been put forward in the literature for using probabilistic transitions to generate SRT sequences. One is that probabilistic transitions allow continuous and flexible assessment of learning progression. Another one is that the probabilistic nature of the sequences allows for a larger variety of sequence chunks to be generated and learned^33,34^.

In both of our experiments, the sequences in the baseline and test blocks are generated from a non-deterministic, first-order Markovian transition matrix between the four instruction keys. In particular, out of all 16 transitions specified between the four keys, the transitions from A to B and C to D are highly probable (*P* = 0.9), and the transitions from B to C and from D to A are medium probable (*P* = 0.7) (see Fig. 1c). In this way, participants often observe reoccurring sequence segments such as AB and CD and could possibly perceive them as “illusory” chunks, even though the generative model is nondeterministic first-order Markovian.

We manipulate the training block sequences across the two experiments. In Experiment 1, three groups of participants were trained on sequences containing no chunks (independent), chunk AB (size 2 chunk), or chunk ABC (size 3 chunk). In Experiment 2, the same “illusory” transition matrix generates the training block sequences but the instructions differ across the two experimental groups. One group is instructed to respond as accurately as possible, while the other is instructed to respond as fast as possible. In order to control for motor effects due to hand and finger dominance, the instructions “A”, “B”, “C”, “D” are randomly mapped to the keys “D”, “F”, “J”, “K” for individual participants. In the next section, we discuss the predictions of our rational model of chunking for the two different experiments and their conditions.

## Related work

Three major types of chunking models have been proposed in the cognitive science literature. The first type are symbolic models, including PARSER and CCN (competitive chunker)^35,36^. Symbolic models learn chunks from already-encountered items and constructs a hierarchy of chunks as participants remember sentences. Sevan-Schreiber and Anderson showed that these models can replicate the behavior of participants’ judgment of grammaticality from sequences with distinct hierarchy levels (e.g., word level vs. phrase level)^36^. Additionally, they replicate the participants’ tendency to overtly chunk the training sentences even when they are presented in an unstructured way. Another model of this kind is PARSER^35^. Proposed by Perruchet and Vinter, PARSER randomly samples the size of the next chunk of syllables and parses the sequence by disjunctive chunks. Each chunk learned by the model is associated with a weight, which increments with observational frequency and decrements via a forgetting mechanism. PARSER can produce artificial language stream segmentations of continuous input streams without episodic cues such as pauses.

The second type are connectionist models of chunk learning. This includes TRACX^37^ and SRN (simple recurrent network)^38^. TRACX uses a three-layer feedforward backpropagation autoassociator and adapts the autoassociator’s weights to the difference between its prediction and actual sequential units when this difference exceeds a pre-defined threshold. Wang et al. trained a self-organized recurrent spiking neural network with spike-timing-dependent plasticity and homeostatic plasticity on sequences. The model was shown to reproduce several sequence learning effects^39^.

The two model types mentioned above are process models. In contrast to process models stand normative statistical models, which model the ideal observers’ behavior. This approach includes variants of the Bayesian ideal observer framework^40^. Given a linguistic corpus, these models find a segmentation with the highest probability that contains relatively few word types, exploiting the minimal description length principle. These models are also rational because their inference is evaluated on observational instances. They provide accounts for high-level computation required for chunk learning. As normative and process models rely on different principles, they are usually not compared against each other.

While these models focused on the benefit of chunking in memory compressibility and grammaticality sensibility, little work relates task instruction with the chunks acquired during learning. Since sequence statistics was the main guidance for chunk learning in these models, instruction modulation has rarely been taken into account.

One typical instruction that changes participants learning behavior is to focus on either speed or accuracy^20^. When task instructions emphasize speed, participants in both lab and naturalistic settings tend to make more mistakes while reacting faster than when instructions emphasize accuracy^21,22^. While earlier work focused on reaction time and accuracy of decision-making tasks^21,23^, little work relates chunking to the speed-accuracy trade-off. It is unclear how instruction will affect chunking and what type of models can take this particular aspect of the task into account.

Here we propose a rational chunking model that takes sequence statistics and task instruction as two parts of a utility function for learning. The model tries to find rational ways of chunking the sequence under task demands, trading off speed with accuracy. Each aspect of the utility function implies a specific prediction: the same task instruction but different sequence statistics should lead to distinct chunking behavior; the same sequence statistics but different instructions should also lead to differently learned chunks. We propose two experiments to test the two aspects of our model’s predictions. For the first experiment, we look at the case when three groups of participants learn from sequences with varying underlying chunks, how chunking changes with varying underlying sequential statistics with different embedded chunks, and show that our model captures participants’ learned chunks. For the second experiment, we look at how participants’ behavior differs when the task instructions focus separately on speed versus accuracy with the same underlying sequence. In doing so, we also propose several novel ways of analyzing RT data based on the speed-up of reaction time, thereby giving insights into how chunks build up across practice trials.

## A rational model of chunking

In the SRT, single instructions *z* out of an instruction set *Z* are presented sequentially. We told participants to press the corresponding key as soon as a new instruction appears. The subsequent instruction shows up in a fixed interval after a participant’s completion of the previous trial. The model learns a set of chunks $C = \{c_1, ..., c_n\}$ and uses the set to parse the sequence. It evaluates the probability *P*(*c*) of parsing each chunk *c* and the conditional probability $P(c_j|c_i)$ that $c_j$ follows $c_i$ for every pair of chunks.

**Figure 2:**
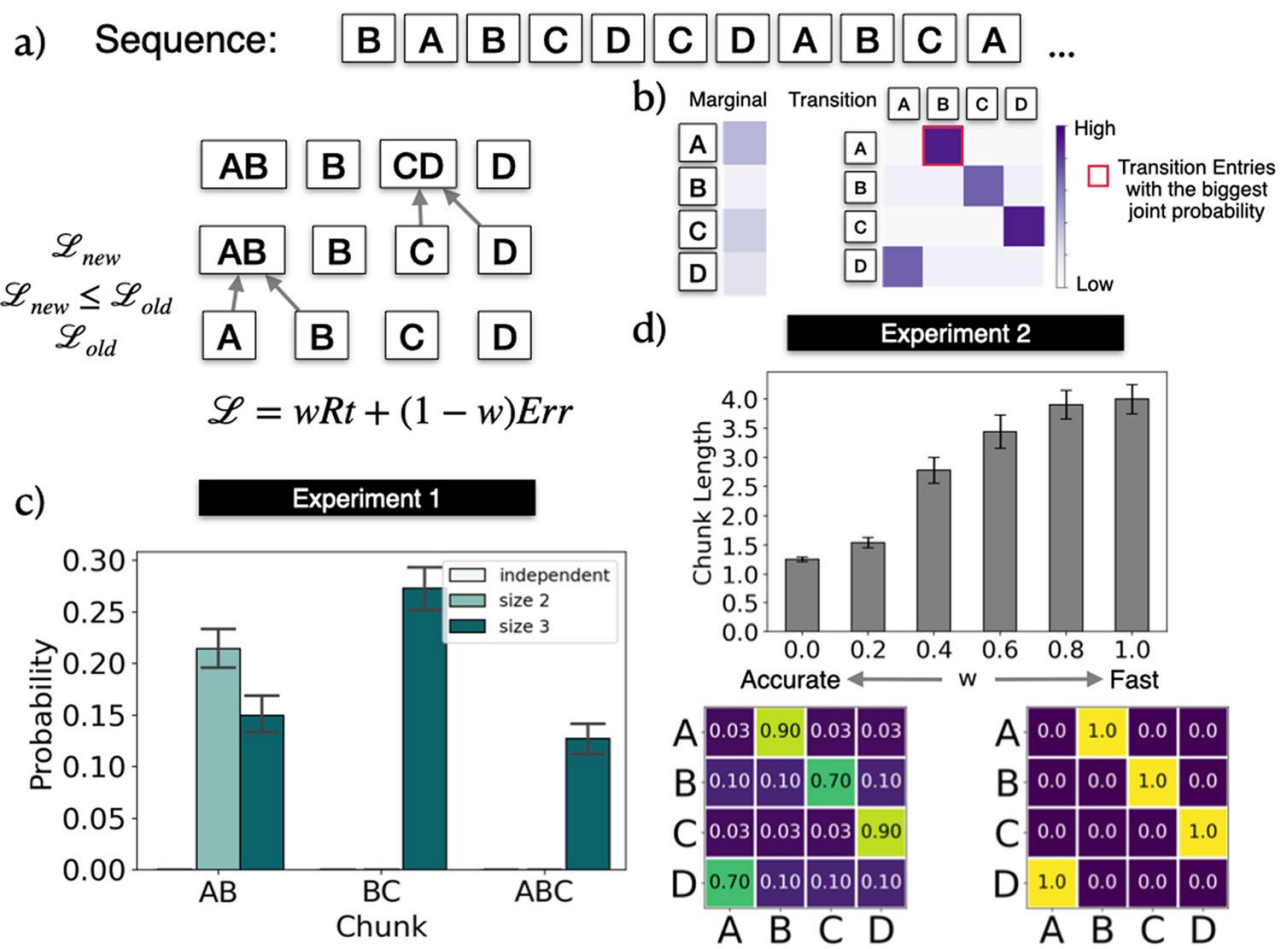
(**a**) Chunking mechanism of rational model. The model keeps track of marginal and transitional probabilities among every pair of pre-existing chunks, and combines chunk pairs that yield the greatest joint probability as the next candidate to be chunked together. At the start, the four different keys are initialized to be the primitive chunks. A loss function that trades off reaction times and accuracy is evaluated on the pre-existing set of chunks. If a chunk update reduces the loss function, then the two pre-existing chunks are combined together. A parameter *w* determines how much more the model weighs an decrease of reaction times compared to an increase in accuracy. (**b**) Example model simulations of learning sequences of Experiment 1. A, B, C, D, are randomly mapped to D, F, J, K for individual participants. Because the transition AB occurred frequently, the model proposes this transition as a possible chunk. (**c**) Model simulation for Experiment 1. Bars represent the probability of a particular chunk parsed in a simulation over the whole experiment. The bars for the independent group on chunk AB, the independent and the size 2 group on chunk BC, and the independent and size 2 group on chunk ABC contain the probability of 0 and are therefore not visible in the graph. Note that these bars can be arbitrarily increased by changing $w$ while the qualitative results remain the same. (**d**) Model simulation for Experiment 2. Top: Average chunk length of different simulations when increasing $w$ from 0 (optimizing only accuracy) to 1 (optimizing only speed). As $w$ increases the average chunk length increases, indicating that the model learns longer chunks when asked to care more about acting fast. Bottom: Transition probabilities learned by model with $w = 0$ and $w = 1$, corresponding to the rational maximization of accuracy and speed. If the model tries to act as accurately as possible, then it recovers the true transition probabilities of the “illusory” transition matrix. If the model tries to act as fast as possible, then sets the medium and high transition probabilities to be 1, i.e. deterministic. All results are averaged across 120 independent simulations. Error bars represent the standard error of the mean.

The set of chunks *C* is initialized as the set of available single instructions *Z* at the beginning of all simulations. The model updates this set by potentially concatenating existing pairs of chunks in *C*. Adding a chunk expands the parsing horizon as the rest of the within-chunk items are predicted to deterministically follow the initiation item of the chunk. Therefore, the subsequent within-chunk items are anticipated in the following trials. The model’s accuracy might diminish if the subsequent instructions are inconsistent with the predicted within-chunk items. We relate subsequent item predictions to reaction times in the next section, and then explain the process by which a rational model updates chunks based on the trade-off between reaction times and accuracy.

### Accounting for reaction times

We use a linear ballistic accumulator (LBA) model to simulate reaction times (RT). LBAs are a common class of multi-choice models^41,42^. In the LBA, each choice corresponds to an evidence accumulator, translated to each four possible key-presses in our task. At every trial of the SRT task, each evidence accumulator starts with an initial evidence $k = \log (P(z_i))$, which reflects the model’s prediction on the upcoming instructions. The trials are divided into within-chunk trials and between-chunk trials. For a within-chunk trial, the prediction for the within-chunk item is the initial evidence for the accumulator $\log (1)$, the rest being $\log (\epsilon )$. Note that the model still integrates information from the SRT instructions but with a high offset which biases it to choose the response which is consistent with the chunk, even if it is inconsistent with the instructed item. This term encourages the model to create longer chunks to reduce the average reaction time.

For a between-chunk trial, the initial evidence for each accumulator $z_i$ is determined by the transition probability $P(c_i|c_j)$ of the chunk $c_i$ that initiates with the accumulator $z_i$, given the previously parsed chunk $c_j$. All response accumulators start from the initial evidence, and drift towards the decision threshold with positive drift rates $v_A, v_B, v_C, v_D$ sampled from a normal distribution with mean $v_{instruction}$ and standard deviation $\sigma$. To simulate the Rt of a particular trial, the current instruction carries the highest drift rate $v_{instruction} = 0.5$ and the evidence accumulators corresponding to the other instructions have an equal but lower drift rate $v_{\lnot instruction} = \frac{1 - v_{instruction}}{3}$. The drift rates for all accumulators sum up to 1. For example, if the current instruction is *A*, then $v_A = 0.5$, $v_B = v_C = v_D = \frac{0.5}{3}$. Evidence accumulation terminates when a positive response threshold *b* is first crossed by any accumulator. The accumulator that crosses the decision threshold first becomes the overt response, and the time it takes to reach the decision threshold is the simulated RT on that trial. In all of the model simulations, we use the same $v_{instruction}=0.5$, decision threshold $b = 1$, $\epsilon = 0.01$, and standard deviation $\sigma = 0.03$ across all accumulators.

### Balancing speed and accuracy

We assume that chunking enables participants to predict upcoming instructions further into the future and thereby to react faster by initializing their evidence at a higher starting point. However, chunking also bears a risk of making mistakes when the upcoming instructions are not the subsequent items within a chunk. We formulate this speed-accuracy trade-off using the loss function

1 $$\begin{aligned} {\mathscr {L}} = w Rt + (1-w) Err , \end{aligned}$$

where $Rt$ is the average reaction time in the SRT, given a learned chunk representation and sequence, and $Err$ is the average error rate. *w* is a parameter that specifies the trade-off between accuracy and reaction time. When $w = 0$, only the reaction time term $Rt$ occupies the loss, and when $w = 1$, the error term $Err$ dominates.

Based on the LBA reaction time simulation, the average reaction time of parsing chunk $c_j$ after previously having parsed the chunk $c_i$ is $\frac{rt_{between}(c_j,c_i)+ (|c_j|-1)rt_{within}}{|c_j|}$. $|c_j|$ is the length of the chunk. The reaction time on the first item is denoted as $rt_{between}(c_j,c_i)$, since $P(c_j|c_i)$ influences the evidence accumulation for this between-chunk key press and only the boundary of the chunk contains transition uncertainty and contributes to the slow down of reaction times. As the initial chunk item determines the chunk identification, the subsequent reaction time to press within-chunk keys in $c_i$ is denoted as $rt_{within}$. This term does not depend on $c_i$ as the procession to the within-chunk items contains no uncertainty. Taken together, the average reaction time can be formulated as follow, averaging the probability of parsing each acquired chunk $c_j$ given the previously parsed chunk $c_i$

2 $$\begin{aligned} Rt = \sum _{c_i \in C}P_C(c_i) \sum _{c_j \in C}P_C(c_j|c_i) \left[ \frac{rt_{between}(c_j,c_i)+ (|c_j|-1)rt_{within}}{|c_j|}\right] , \end{aligned}$$

Similarly, if we formulate $R_{LBA}(z_j)$ as the response choice of the LBA model when the instruction is $z_j$, then we can denote an error occurrence as $\mathbbm {1}\left[ z_j \ne response(z_j)\right]$, which is an indicator function that becomes 1 when the instruction $z_j$ is inconsistent with the LBA response. The average error rate can be evaluated by averaging the error rate with the probability of single-element transitions from the generative model $P_I$, enabling the formulation of the expected error rate as

3 $$\begin{aligned} \text {Err} = \sum _{z_i \in \{A,B,C,D\}}P_I(z_i) \sum _{z_j \in \{A,B,C,D\}}P_I(z_j|z_i) \mathbbm {1}\left[ z_j \ne R_{LBA}(z_j)\right] \end{aligned}$$

This utility function, therefore, induces a trade-off between being accurate (predicting elements correctly) and being fast (finding a chunk representation to predict further ahead and speed up one’s reaction time). Together, these parts of the loss are used to evaluate the utility of a chunk representation under specific task demands.

### The rational update of chunking

The model updates the chunk representation rationally by concatenating chunks within the chunk set *C* that induce a lower loss. *C* is initialized with single sequential items $\{A,B,C,D\}$. For one set of chunks *C*, the model evaluates the marginal probabilities of each chunk $P_C(c_i), c_i \in C$ and the transition probability $P_C(c_j|c_i)$ of parsing chunk $c_j$ after having parsed chunk $c_i$. $P_C(c_i)$ and $P_C(c_j|c_i)$ are stored in the marginal and transition probability matrices as shown in Fig. 2b. The marginal and transition probability is evaluated empirically over an entire sequence parse using chunks in *C*.

We can calculate the joint occurrence probability of concatenating chunk $c_i$ with $c_j$ as $P(c_i, c_j) = P_C(c_j|c_i) P_C(c_i)$. The chunk pair $c_i$, $c_j$ with the highest joint probability is suggested as a new chunk to replace $c_i$ to form a new to the set of chunks $C_{new}$. As the initiation of $c_i$ is predictive of the subsequent chunk items. For example, an addition to $\{A, B, C ,D\}$ could be a new chunk *AB*. The new chunk *AB* then replaces *A* and the new proposed set of chunks $C_{new}$ becomes $\{AB, B, C, D\}$.

We then compute whether $C_{new}$ is accepted to replace the original set of chunks *C*. The acceptance depends on whether the new set of chunks $C_{new}$ and the induced reaction time in addition to the marginal and transition probabilities upon parsing the sequence lead to a lower loss. In case it is so, $C_{new}$ replaces *C*, which becomes the basis of proposing the next chunk. This chunk proposal process continues until a fixed iteration number, as shown in Fig. 2a.

### Model predictions for experiment 1

We first examined the model’s chunk learning behavior on the three groups of Experiment 1. In this simulation, the underlying generative model either contained no chunks (independent), the chunk AB (size 2 chunks), or the chunk ABC (size 3 chunks). We then fixed the trade-off parameter $w$ to optimize accuracy more than speed by setting it to $w = 0.2$. Figure 2c shows the probability of chunk AB, BC, and ABC being learned as subchunks by the rational model of chunking over 120 simulations in total. The model uses the entire sequence to learn its chunk representation in each simulation. With the same trade-off between speed and accuracy, the rational chunking model trained on sequences with size 2 and 3 chunks has a higher probability of learning AB as a subchunk than a model trained on the independent sequence. Chunk BC has a higher probability of being learned by the model trained on sequences containing size 3 chunks than models trained on sequences with independent instructions or sequences with size 2 chunks. Only the model trained on sequences containing size 3 chunks learned about the chunk ABC. Taken together, these simulations predict that participants in the different conditions will be more likely to learn the corresponding chunks than participants for whom a chunk is not part of the training sequence.

### Model predictions for experiment 2

We examined the model’s chunk learning behavior for Experiment 2. According to our model, changing the trade-off between accuracy and speed translates to changing the cost function’s $w$ away from 0 and towards 1. We therefore simulated the behavior of our model with changing $w$ (Fig. 2d). As $w$ goes from 0 to 1, i.e. the cost function shifts from minimizing the model’s error rate to minimizing its reaction time, the average length of chunks learned by the model increases. Thus, our model predicts that participants in the fast group, which demands speedier responses, should learn longer chunks as compared to participants in the accurate group. Evaluating the single-element transition probability with $w = 0$ and 1 (Fig. 2d) shows that if only accuracy is the optimization goal of the cost function, then the model preserves the original transition matrix. However, if the model optimizes for speed, then it learns a polarized transition probability where all the high and medium single element transitions attract more probability mass, i.e. are closer to 1. Correspondingly, the remaining probabilities are closer to 0. Thus, as the high and medium transitions are more integrated into the chunks, this gives the model a speed-up in its reaction times, because it can start its evidence accumulation at a higher initial point. This comes at the cost of accuracy, because the initialization may be incorrect.

## Experiment 1: learning about true chunks

In Experiment 1, we test the model’s prediction that chunking behavior adapts to the statistics of the sequence. When chunks are used to generate the sequence, participants should learn more than those trained on sequences without chunks.

Experiment 1 was conducted using a between-groups designs in which 122 participants were randomly assigned to one of three groups at the beginning of the experiment. These groups were the independent, size 2, and and size 3 conditions. The experiment was comprised of 10 blocks in total. The middle six blocks were the training blocks where participants practised the independent, size 2 or size 3 sequences. The first two and the last two blocks were the baseline and test blocks. In those blocks, all three groups of participants received the same sequence generated from the “illusory” transition matrix. Training blocks differed amongst the three groups, as shown in Fig. 1, while the baseline and the test blocks remained the same. For the training blocks, the independent group practiced sequences that contained no chunks, the size 2 group practiced sequences with chunk AB, and the size 3 group practiced sequences with chunk ABC, as shown in Fig. 1d. The sequence for the independent group was randomly and independently sampled from single-item elements A, B, C, and D with equal probability. This means that this sequence contained no chunks. The sequence for the size 2 group was generated by sampling AB, C, and D with an equal probability of 1/3. In other words, this sequence contained the chunk AB. The sequence for the size 3 was generated by sampling ABC and D with an equal probability of 1/2. Thus, this sequence contained the chunk ABC. All three groups received the same instruction to act “as fast and accurately as possible” throughout the experiment. On each trial, participants received feedback on the reaction time and correctness of the previous trial, followed by a 500ms response-to-stimulus interval. Participants were informed that their performance bonus on top of a base-pay was based on a mixture of their reaction times and accuracy. The baseline and test blocks were sequences generated by the illusory transition matrix in Fig. 1c. The main prediction was that if people have learned chunks present in the training blocks, then they will use them even in the test blocks. We measured this by examining differences in accuracy and reaction times from the baseline block. We also used Experiment 1 to validate several of our empirical measures of chunking which we will use in Experiment 2.

We decided to examine model prediction on multifaceted prospects of participants’ chunk learning behavior by proposing and applying various chunk learning measures at distinct stages of the task. Since many aspects of the measure are novel, we conduct these measures in the hope that their results complement each other.

The first two measures, chunky boost, and chunkiness, evaluate indicators of learning size 2 and size 3 chunks by comparing the performance of the baseline and test blocks. The regression on chunky RT evaluated on the test block examines the transition probability’s influence on reaction time. The last three measures rely on chunks as identified by the mixture of the Gaussian method. They are directly-measured from the chunking profile of the participants. The chunk growth rate evaluates chunk size increase during training. The chunk increase measure shows the quantitative differences between counted chunks between the baseline and the test blocks. The last measure on chunk reuse probability looks for the character of reusing previously learned chunks to construct new chunks, as demonstrated by participants’ chunk learning.

**Figure 3:**
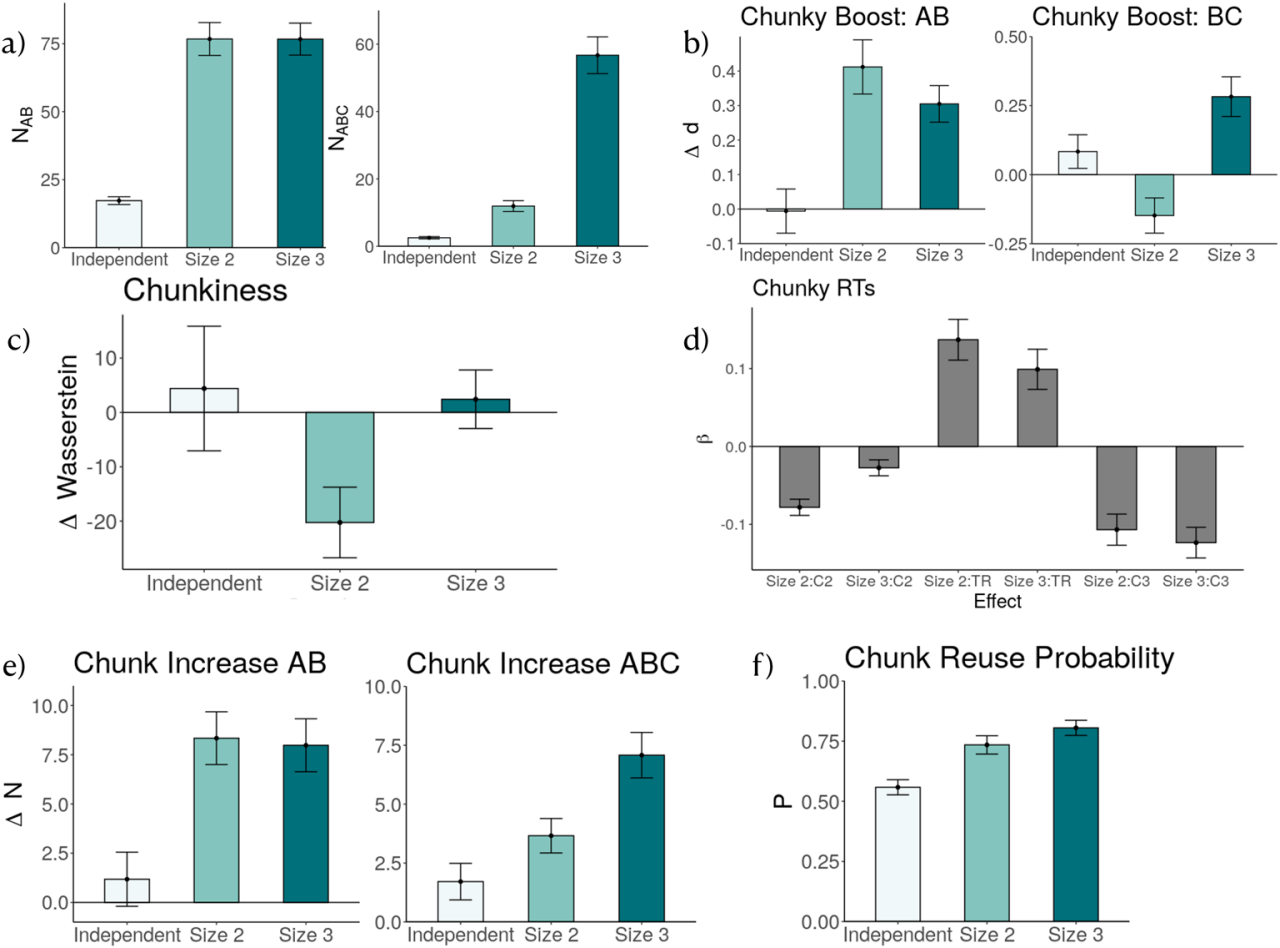
Results of Experiment 1. (**a**) Manipulation check. The number of chunks AB and ABC learned by participants during the training blocks by group. Chunks were retrieved using a categorization of between- and within-chunk transitions by a mixture of Gaussians analysis of participants’ reaction times. (**b**) Chunky Boost of size 2 chunks AB and BC by group. A chunky boost is measured by the relative change of Cohen’s *d* between baseline and test blocks for the highly and medium probable transitions. (**c**) Chunkiness of size-3 chunks ABC. Chunkiness is measured by the relative change of Wasserstein distance between the baseline and test blocks of between-chunk reaction times of all possible size 3 chunks. (**d**) Regression coefficients of interaction effects between condition and size 2, size 3, and true transition probabilities on reaction times during the final test blocks. (**e**) Chunk increase from the baseline to the test blocks by group for chunk AB and chunk ABC. Chunk increase is measured by the number of returned chunks from the mixture of Gaussians analysis. (**f**) Chunk reuse probability by group. Chunk reuse probability was calculated based on whether or not part of an earlier chunk were used in a later chunk that occurred within the next 30 trials. For all plots, error bars indicate the standard error of the mean.

### Manipulation check

We first checked if participants’ behavior during the training blocks reflected the underlying chunks in the generative model. In particular, we tested whether the size 2 group showed evidence for learning chunk AB, and the size 3 group learning chunk ABC. We used a Gaussian mixture model to categorize reaction times of each response from the same participant into fast “within” or slow “between” chunk transitions, based on the assumption of a within-chunk speed-up. This method gave us a glimpse into how the action sequence was partitioned by participants, reflecting their internal representation of chunks (more details in Methods). We then counted the number of times chunks AB and ABC showed up in the training block, denoted as $N_{AB}$ and $N_{ABC}$. If the size 2 group and the size 3 group had learned chunk AB and ABC, separately, then $N_{AB}$ should be higher for these two groups than the independent group, and $N_{ABC}$ should be higher for size 3 group than the other two. Figure 3a shows the average $N_{AB}$ and $N_{ABC}$ returned by this analysis across the three conditions during the training blocks.

For $N_{AB}$, fitting a linear regression model using condition as the independent variable and the number of chunks $N_{AB}$ as the dependent variable showed a significant effect of condition ($F(2) = 45.02$, $p < 0.001$). $N_{AB}$ was higher for both the size 2 group ($\hat{\beta } = 59.43$, $t(139) = 8.20$, $p < 0.001$) and the size 3 group ($\hat{\beta } = 59.39$, $t(139) = 8.32$, $p < 0.001$) than for the independent group. This means that training on sequences that contained either AB or ABC chunks induced participants to learn AB as a chunk.

To investigate differences in the acquisition of the ABC chunk between groups, we repeated the same regression with $N_{ABC}$ as the dependent variable. We found a significant effect of groups ($F(2) = 71.45$, $p < 0.001$), indicating that participants’ responses reflected ABC chunks more often in both the size 2 ($\hat{\beta } = 9.40$, $t(139) = 1.89$, $p = 0.06$) and size 3 ($\hat{\beta } = 54.17$, $t(139) = 11.07$, $p < 0.001$) than in the independent group. Interestingly, we observed higher $N_{ABC}$ with the size 2 group than in independent group. This can be because building on top of a previously learned chunk (AB $\rightarrow$ ABC) is more accessible for the size 2 group than the independent group (as the independent group needs to learn chunk AB first, then ABC). Furthermore, $N_{ABC}$ was significantly higher for the size 3 than the size 2 group ($\hat{\beta } = 44.76$, $t(139) = 9.25$, $p < 0.001$), suggesting that training on sequences that contained ABC chunks resulted in the strongest tendency of participants to learn ABC as a chunk.

Given these results, we conclude that our experimental manipulation of the three groups induced the intended behavior during the training blocks.

### Chunky boost

When trained on sequences with underlying chunks ABC and BC, the rational chunking model learns chunk ABC and BC separately. To check this prediction of our model, we look at participants learning of size 2 chunks, i.e. AB and BC, separately. In particular, we look at participants’ reaction time of pressing within chunk items, B in AB and C in BC, and how these items speed up differently across the three groups from the baseline to the test blocks. In SRT tasks, the reaction time difference before and after training is usually used as a sensitive measure of skill^25^. If participants’ behavior is consistent with our model’s prediction, then the size 2 and size 3 groups should have a stronger sign of learning chunk AB than the independent group. The size 3 group should have a stronger sign of learning chunk BC than the size 2 group.

We look at how the training schedule changes the value of within-chunk (value marked by the red boundary) reaction time for AB and BC (since a sign of chunking is that the reaction time of within-chunk items is typically faster than between-chunk items^8,12,43^); a figurative explanation of this method can be found in Fig. 6 in the appendix. We look at the within-chunk reaction time of AB and BC for all groups at the baseline and the test blocks and compute the difference by the signed effect size, Cohen’s d, of the baseline blocks, compared to the test block $d_{AB}$. Cohen’s d is a standardized measure of how far the means of two probability distributions are apart. In this case, these two distributions are the reaction time in the baseline blocks and the reaction time in the test blocks. We used a signed version of Cohen’s d to convey the relative change of the reaction time distributions. $d_{AB}$ is positive when, on average, the reaction time of B in AB at the test block is faster than the reaction time in the training block – a sign of learning. However, solely looking at AB and BC is not enough, as a general learning factor will speed up participants’ reaction time naturally. Therefore we compared the signed effect size AB and BC with the reaction time speed up of the control chunks. For the controlled between-chunk items, we evaluated the signed Cohen’s d on AA, AB, and AC for chunk AB; and on BA, BB, and BD for chunk BC. Finally, we arrived at the chunky boost measure $\Delta d$ by subtracting the relative speed-up of AB and BC from their corresponding control chunks. We named this a chunky boost measure. Figure 3b shows the Chunky Boost of AB and BC across the three groups.

For chunk AB, fitting a linear model onto participants signed Cohen’s d change showed a significant effect of group ($F (2) = 10.613$, $p < 0.001$); participants in the size 2 group had a higher relative change of Cohen’s d than the independent group ($\hat{\beta } = 0.41$, $t = 4.41$, $p < 0.001$). Thus, training on the chunks with size 2 made the size 2 group respond to B faster after having seen item A. Additionally, participants in the size 3 group also had a higher relative change of reaction times responding to chunk AB than the independent group ($\hat{\beta } = 0.31$, $t = 3.35$, $p = 0.001$), showing that their reaction to B also sped up relative to control. These results are consistent with the model prediction that chunk AB should be acquired by the size 2 and size 3 group, separately.

For chunk BC, fitting a linear model onto the chunky boost measure $\Delta d$ on BC with group as the independent variable also showed a significant effect ($F (2) = 10.802$, $p < 0.001$). Interestingly, the size 2 group had a negative chunky boost to BC ($\hat{\beta } = -0.23$, $t = -2.44$, $p = 0.02$), showing a relative reaction time slow-down compared to control. This effect was expected because identifying B as the end of a chunk will result in the transition to C as a “between-chunk” transition. In other previous SRT experiments, a slow-down in between-chunk reaction times was also observed^44^. This slow-down can contribute to the negative chunky boost of the size 2 group. Relative to the independent group, the size 3 group had a significantly higher chunky boost $\Delta d$ ($\hat{\beta } = 0.20$, $t = 2.14$, $p = 0.03$). This shows that learning chunks changes the reaction time profile of this group. Their response to C upon previous instruction B was speeding up their reaction times much more from the training blocks to the test blocks compared to control. This is consistent with the model prediction that the size 3 group should be more likely to learn chunk ABC.

In summary, participants’ reaction times changed in a predictable fashion, with the independent group not getting faster for either AB or BC, the size 2 group becoming faster for AB and slower for BC, and the size 3 group becoming faster for both AB and BC. These observations confirmed previous work studying chunking in SRT tasks, which has argued that RTs in structured sequences decrease more quickly than in non-structured sequences^12^ and are consistent with the predictions from the rational chunking model.

### Chunkiness

The rational chunking model predicts that the size 2 group should learn more chunks AB, and the size 3 group should learn more chunks ABC, compared to the independent group. To access this prediction, we formulated a measure of chunkiness as an indicator of learning size 3 chunks. If participants have learned a size 3 chunk, such as ABC, then the distributions of within-chunk reaction times (i.e. the reaction time of B and C) should become more similar to each other^8^. We use the Wasserstein distance to evaluate the homogeneity of reaction time distribution of B and C, $rt_B$ and $rt_C$, following the presentation of A. The Wasserstein distance is also known as the “earth mover’s” distance. It can be seen as the minimum amount of “work” required to transform one distribution into another. “Work” is the amount of distributional weight that must be moved multiplied by the distance (see also Supporting Information). This is simply just measuring how similar the two reaction time distributions are.

We evaluated the Wasserstein distance between the distribution of $rt_B$ and $rt_C$ on the baseline blocks, when all groups of participants are trained on the illusory transition sequences, to arrive at $Wasserstein(rt_B, rt_C)_{baseline}$. This assesses the initial separation of the two distributions, how participants learn from the illusory transition sequences, when all groups of participants have not been exposed to any training that involves chunks. Then we evaluate the same Wasserstein distance in the test blocks, also during the illusory transition sequence, to arrive at $Wasserstein(rt_B, rt_C)_{test}$, to assess how much training influences $rt_B$ and $rt_C$ in the test blocks. If participants have learned ABC as a chunk during the training blocks, then $rt_B$ and $rt_C$ should become more homogeneous in the test blocks, resulting in a smaller Wasserstein distance, as compared to the baseline blocks. We subtracted $Wasserstein(rt_B, rt_C)_{test}$ from $Wasserstein(rt_B, rt_C)_{baseline}$ to calculate this change of reaction time homogeneity: $\Delta W_{chunk}$. This relative change of Wasserstein should be positive if reaction times became more homogeneous in the test blocks. Since training may result in an overall increase of reaction time homogeneity for all items, we compared this change of $Wasserstein$ with size 3 sub-sequences that were not ABC, as a control. $\Delta W_{control}$ as the difference between $W_{test}$ and $W_{train}$ is evaluated on the control sequences.

Finally, we subtract $\Delta W_{control}$ from $\Delta W_{chunk}$, to arrive at the resulting measure of “chunkiness”. Chunkiness can be seen as the relative change of the Wasserstein distance of chunk ABC $\Delta W_{ABC}$ compared to control $\Delta W_{control}$. The resulting evaluation of chunkiness on the three groups is shown in Fig. 3c. Chunkiness differed significantly between the three conditions ($F(2) = 3.20$, $p = .04$).

In particular, the size 2 group had a negative relative Wasserstein shift ($\hat{\beta } = -26.92$, $t(137) = -2.37$, $p = 0.02$). This means that the size 2 group’s reaction time distribution became less homogeneous after training, indicating that the reaction time to press B deviated more from C. This was expected as for the size 2 group, pressing B and C after A should be one within and one between-chunk reaction time. On the other hand, the change of Wasserstein distance between the size 3 and the independent group condition was not significant, even though we would have expected this group to become more homogeneous in their reaction times. One reason for this surprising result could be that, while the reaction time distribution upon the instructions “B” and “C” became closer to each other relative to control, the shift may have not uniformly impacted the calculation of Wasserstein distance. It could also be that positions at the end of a chunk can be learned faster than the intermediate elements, as found in^45^.

In summary, we verified the prediction that the size 2 group had less homogeneous transition times within the chunk ABC than the other groups. However, we did not observe an increased chunkiness for the size 3 group, possibly due to non-uniform speed-ups of RTs.

### Reaction time regression

The learning of chunks during the training blocks will influence how participants perceive the transition from one instruction to another. As the rational chunking model predicts that participants in the size 3 group will learn chunk ABC, size 2 group will learn chunk AB, and no such chunks in the independent group. This chunk learning will influence how participants perceive the items in the test blocks in a way that size 2 group and size 3 group may react to the sequence in a more deterministic manner. Therefore we studied the influence of transition probabilities on participants’ reaction times during the test blocks (Fig. 3d) on the correct trials. We use three transition probability matrices as regressors. One is the true transition (TR), which is the ground truth transition probability used to generate a sequence in the test block. The second one is C2 transition matrix that contains a deterministic transition from A to B. And the third one is C3 transition matrix, with a deterministic transition from A to B, and B to C. The rest of the entries of C2 and C3 are the same as TR.

We fitted a linear mixed-effects regression using log-reaction times as the dependent variable, assuming a random intercept for each participant. The independent variables were the TR, C2, and C3 transition probabilities, group, as well as interaction effects between group and each of the transition probabilities.

The best regression contained the predicted transition probabilities as well as three interaction effects with groups ($\chi ^2(8) = 129.6$, $p < 0.001$). The first interaction was between TR and the size 2 group ($\hat{\beta } = 0.13$, $t(25040) = 5.24$, $p < 0.001$), showing that the effect of TR learned by the size 2 group was significantly up-weighted. The interaction between TR and the size 3 group was also significantly up-weighted ($\hat{\beta } = 0.10$, $t(25040) = 3.84$, $p <0.001$). TR transition probabilities as an independent variable slowed down the reaction times for the size 2 and size 3 groups more than for the independent group.

The interaction was significantly down-weighted between the C2 chunky transition probabilities and the size 2 group ($\hat{\beta } = -0.08$, $t(25040) = -7.51$, $p < .001$) and the size 3 group ($\hat{\beta } = -0.03$, $t(25040) = -2.68$, $p = 0.007$). This indicates that C2 transition probabilities sped up the reaction times for the size 2 and the size 3 group more than for the independent group, and the effect is stronger for the size 2 group.

Finally, the interaction was down-weighted between the C3 transition probabilities and the size 3 group ($\hat{\beta } = -0.12$, $t(25040) = -6.27$, $p < .001$), as well as an interaction between the C3 transitions and the size 2 group ($\hat{\beta } = -0.11$, $t(25040) = -5.34$, $p <0.001$). These significant interaction effects indicate that C3 transition probabilities sped up the reaction times for the size 2 and the size 3 group more than that for the independent group. In summary, we found predictable relations between participants’ reaction times and the chunk-implied transition probabilities across groups. In particular, C2 transitions were more significantly related to speed-ups for the size 2 group than the size 3 group, while C3 transitions significantly related to speed-ups for the size 3 group more than size 2 group. This relation is consistent with the prediction generated by the rational chunking model.

### Chunk increase

The rational model of chunking, as shown in Fig. 2c, predicts that participants’ learned chunks should reflect the underlying chunks used to generate the sequence. That is, the chunk 3 group should learn more chunk ABC than the chunk 2 group and the independent group. Additionally, both chunk 3 and chunk 2 group should learn more chunk AB than the independent group. This acquisition of chunks during the 6 training blocks is going to influence how participants behave in the baseline and test blocks. We here test this prediction concretely by examining how often chunk AB and chunk ABC are used by the three groups in the test blocks, using baseline blocks as a control.

We look at the exact chunks used by participants by classifying within and between chunk reaction time using the mixture of Gaussian method (see section method). As an illustration, Fig. 5 shows the participants’ data. The reaction time, instruction displayed, and this participant’s actual key press is shown on the first, second, and third row. Instruction for A, B, C, D, is separately color-coded by green, blue, magenta, and orange boxes. When this participant has pressed a key incorrectly, that trial is marked by a red box. Using the distribution of reaction time data accumulated for this participant over all trials, we classify individual trials into within or between chunk key press, whichever results in a higher likelihood in the mixture. Once each trial is classified as within or between chunk trials, we can mark the chunks learned by this participant by connecting all within-chunk trials and the first between chunk trial that starts before those within-chunk trials as belonging to the same chunk (thereby, we can mark the size of chunks by connecting black round dots shown on the fourth row of the above). In this way, we can identify the content of the chunks learned by this participant as reflected by their reaction time speed-up, in addition to the chunk size learned by each participant, as illustrated in Fig. 5 in supplementary information.

We measured the number of times each participant chunked AB and ABC in baseline and test blocks and evaluated the increase $\Delta N = N_{test} - N_{baseline}$. Figure 3e shows $\Delta N_{AB}$ and $\Delta N_{ABC}$, measured separately for the three groups.

Fitting a linear model setting $\Delta N$ of AB as the dependent variable and group as an independent variable showed a significant effect of group ($F(2) = 8.64$, $p < 0.001$). Compared to the independent group, the size 2 group ($\hat{\beta } = 7.16$, $t(139) = 3.68$, $p < 0.001$) and the size 3 group ($\hat{\beta } = 6.80$, $t(139) = 3.55$, $p < 0.001$) chunked AB significantly more often in the test blocks than in the baseline blocks.

The same analysis for $\Delta N$ of chunk ABC also showed a significant effect of group ($F(2) = 10.63$, $p < 0.001$). The size 3 group chunked significantly more ABC as chunks than the independent group ($\hat{\beta } = 5.36$, $t(139) = 4.53$, $p < 0.001$). Participants in the size 2 group also chunked more ABC than the participants in the independent group ($\hat{\beta } = 1.94$, $t(139) = 1.62$, $p = 0.10$). Compared with the size 2 group, the size 3 group chunked ABC also significantly more often ($\hat{\beta } = 3.42$, $t(139) = 2.92$, $p = 0.004$). Overall, participants’ behavior is qualitatively consistent with model prediction. Training on sequences with chunks increased participants’ tendency to use those chunks in the test blocks.

### Chunk reuse

Since the rational chunking model reuses previously learned chunks to construct new ones, we wanted to study whether participants’ chunking behavior reflected this feature of our model. As explained in the method section, the mixture of the Gaussian rt classification method returns the estimated learning progress of chunks for each participant throughout the experiment. We examined participants’ chunk reuse probability based on how individual participants learned the chunks during the training blocks (Fig. 3f).

The chunk reuse probability was evaluated on chunks of size three or bigger (excluding chunks with single-item repetitions). Every time such a chunk occurs in the sequence, we check whether it reuses any of the 30 previous chunks. Figure 8 in SI shows an example of chunk CDABCD reusing BCD as one of its previous chunks. By tagging each chunk learned by the participant as reusing one of the previous chunks or not, we arrived at a chunk reuse probability for each participant. Figure 3f shows the average chunk reuse probability across the three groups.

We found that the chunk reuse probability differed significantly between the groups. Fitting a linear model taking the reuse probability as the dependent variable and group as the independent variable showed a significant effect of condition ($F(2) = 13.99$, $p < 0.001$). Both the size 2 ($\hat{\beta } = 0.17$, $t(139) = 3.63$, $p < 0.001$) and the size 3 group ($\hat{\beta } = 0.24$, $t(139) = 5.17$, $p < 0.001$) reused chunks significantly more often than the independent group. There was no significant difference between the size 2 and the size 3 group ($\hat{\beta } = 0.07$, $t(139) = 1.51$, $p = 0.13$). The tendency to reuse previously learned chunks is consistent with how our model creates chunks, i.e., creating a new chunk by combining previously learned chunks. Interestingly, sequence statistics modulated participants’ tendency to reuse chunks. When the sequence contained embedded chunks that render reuse beneficial to performance, participants tended to reuse previously acquired chunks more often than when the sequence only contained independent item instantiations. The observation that participants reused previously acquired chunks echoes previous findings in the literature on transferring motor skills, which showed that people transfer chunks from a practiced sequence to a test sequence when shared chunks between the two^14^. The reuse and transfer process in the current task was an ongoing learning behavior while participants practiced the training sequence.

## Experiment 2: learning chunks of different sizes to balance the speed–accuracy trade-off

In Experiment 2, we test the model prediction that chunk learning adapts to task demands. Participants should chunk more under time pressure, even given a sequence without chunks within.

We randomly assigned participants to one of two groups: the fast group and the accurate group, creating a two-groups between-subjects design. Both groups were trained on sequences generated from the “illusory” matrix that contained no true chunks but high and medium single item transitions (Fig. 1c). The experiment structure was identical to the structure of Experiment 1: 10 blocks with 100 trials each. The training blocks were sandwiched between baseline and test blocks, see Fig. 1a. In those blocks, accuracy and reaction times were displayed right at the end of each trial. In the middle 6 blocks, from block 3 to block 8 (i.e. the training blocks), participants in the fast group were instructed to act “as fast as possible even if it might lead to mistakes”, and participants in the accurate group were instructed to act “as accurate as possible even it might slow you down”. The fast group was told that their reward depended on how fast they pressed the instructed key and were given trial-by-trial feedback on their reaction times. The accurate group was told that their reward depended on their accuracy and were given trial-by-trial feedback on the correctness of their responses. Both groups received the same instruction to act “as fast and accurately” as possible during the baseline and the test blocks (block 1-2 and 9-10, see Fig. 1a.

**Figure 4:**
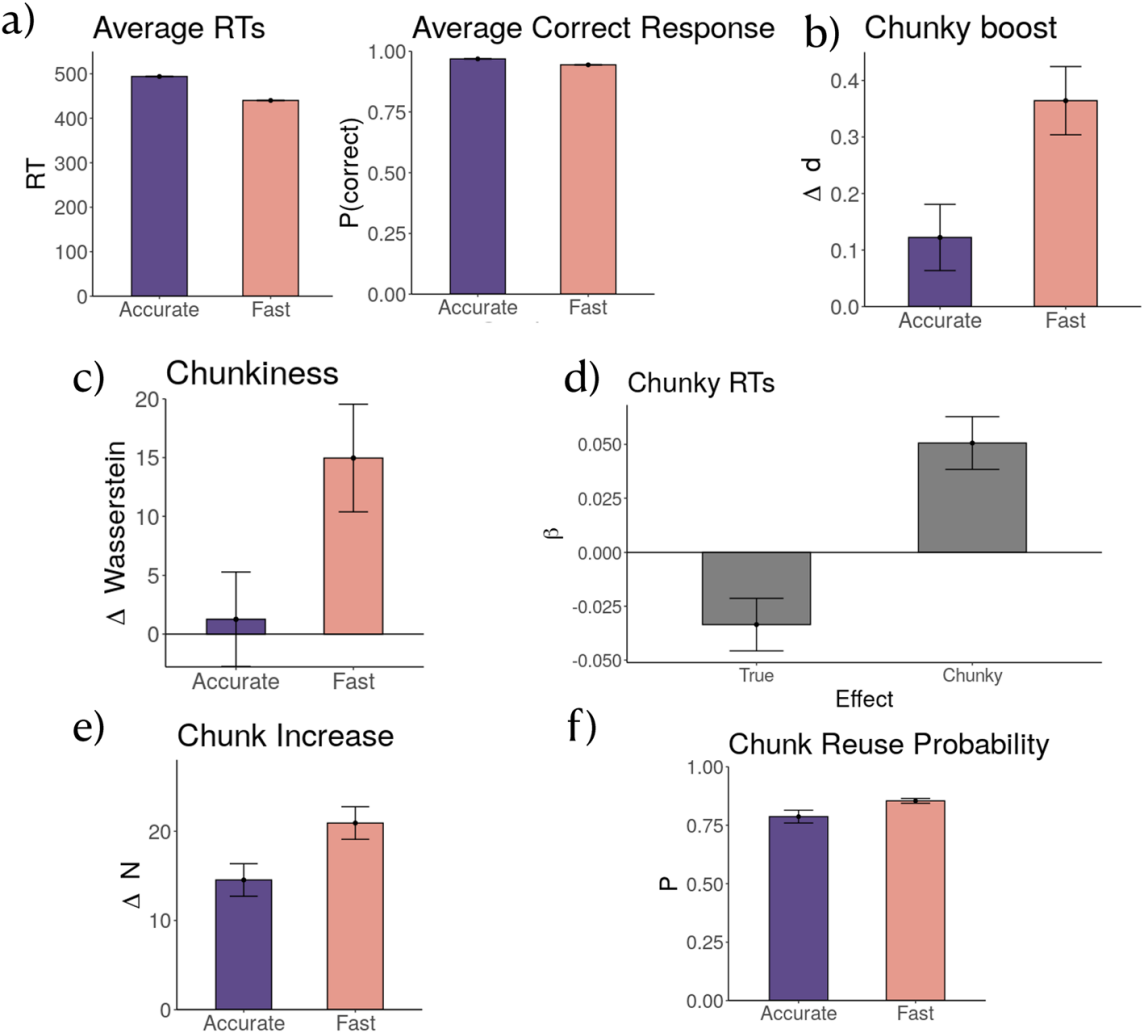
Results of Experiment 2. (**a**) Manipulation check. Average reaction times and average response accuracy during training blocks by group. (**b**) Chunky Boost of size 2 chunks as measured by change of Cohen’s *d* by group evaluated on baseline and test blocks. The size 2 chunks include AB, BC, CD, and DA. (**c**) Chunkiness measured by a relative change of Wasserstein distance of size 3 chunks including ABC, BCD, CDA, DAB between the baseline and the test blocks. (**d**) Coefficient of interaction effect between chunky and true transition probabilities on reaction times during the test blocks. (**e**) Chunk increase from the baseline to the test blocks by condition for size-2 (AB, CD, BC, DA) and for size-3 chunks (ABC, BCD, CDA, DAB). (**f**) Chunk reuse probability by group. For all plots, error bars indicate the standard error of the mean.

### Manipulation check

We first assessed whether the instructions to be fast or accurate influenced participants’ reaction times and accuracy during the training blocks. Shown in Fig. 4a are the average reaction time and accuracy for the two groups. Fitting a linear mixed-effects regression onto participants’ reaction times assuming a random intercept over individual participants showed a significant effect of group ($\chi ^2(1)=9.84$, $p=.002$), showing that participants in the fast group responded faster during the training blocks than participants in the accurate group ($\hat{\beta }=81.71$, $t(113.93)=3.19$, $p=.0001$). We also fitted a mixed-effects logistic regression of group to test whether participants responded correctly on each trial, adding a random intercept for each participant. This analysis also showed a significant effect of group ($\chi ^2(1)=9.67$, $p=.002$), with participants in the accurate group responding on average more accurately during the training blocks than participants in the fast group ($\hat{\beta }= 0.54$, $z=3.18$, $p=.001$). Thus, we conclude that our experimental manipulation induced the intended behavior for the two groups during the training blocks.

### Chunky boost

The rational chunking model predicts that the fast group, compared to the accurate group, should learn more chunks. This influence of different instructions will affect the behavioral change of both groups’ performance in the test blocks relative to the baseline blocks. We again look at an indicator of learning size-2 chunks by evaluating chunky boost on the within-chunk reaction times of the size 2 chunks. This time, the chunky boost was evaluated on the most frequently occurring size-2 chunks in the sequence produced by the “illusory” transition matrix: AB, BC, CD, and DA. AB and CD are the size-2 chunks with high transition probability ($p = 0.9$). BC and DA are size 2 chunks with medium transition probability ($p = 0.7$). The corresponding control chunks were size two subsequences that did not begin with the first chunk items. As an example, the control chunks for AB were BB, CB, and DB.

We conjectured that the fast group would learn more size two chunks with high and medium probability. We look at how the training schedule changes the value of within-chunk (value marked by red boundary) reaction time for size 2 chunks in the test blocks compared to the baseline blocks. We calculate the signed effect size, Cohen’s d, on the within-chunk reaction time from the baseline to the test block. The same procedure was applied for the control chunks. Then the Cohen’s d of the control chunks was subtracted from the size 2 chunks to arrive at the chunky boost measure. The chunky boost measured by a change of Cohen’s d $\Delta d$ is shown in Fig. 4b. Fitting a linear mixed-effects regression onto participants’ change of Cohen’s d, assuming a random intercept over participants showed a significant effect of the group ($\chi ^2(1) = 7.25$, $p = .007$). Participants in the fast condition showed a greater relative boost in reaction times to chunky transitions as compared to participants in the accurate group ($\hat{\beta } = 0.24$, $t(73) = 2.71$, $p = .008$). We, therefore, concluded that participants in the fast group chunked more size two chunks than participants in the accurate group, as was predicted by our model.

### Chunkiness

The rational chunking model that exerts a speed-accuracy trade-off predicts that the fast group should learn longer chunks than the accurate group. Similar to the analysis in experiment 1, we examined this prediction from the model by evaluating the chunkiness measure as an indicator of participants learning size-3 chunks (ABC, BCD, CDA, DAB) that can occur in the training sequence generated by the “illusory” transition matrix. If participants have learned any of those size-3 chunks, then the distributions of within-chunk reaction times $rt_2$ and $rt_3$ should become more similar to each other following the presentation of the first item. We use the Wasserstein distance to evaluate the homogeneity of this reaction time distribution, illustrated in Fig. 7.

To assess the initial separation of the two distributions for both groups before training, we evaluated the Wasserstein distance between the distribution of $rt_2$ and $rt_3$ on the baseline blocks, $Wasserstein(rt_2, rt_3)_{baseline}$, when both groups of participants are trained on the illusory transition sequences. To assess how much training influences $rt_2$ and $rt_3$ in the test blocks, we evaluate the same Wasserstein distance on both groups in the test blocks, to arrive at $Wasserstein(rt_2, rt_3)_{test}$. An indicator of learning the frequent size-3 chunk (ABC, BCD, CDA, or DAB) during the training blocks, is that $Wasserstein(rt_2, rt_3)_{test}$ should become smaller in the test blocks than in the baseline blocks, resulting in more homogeneous $rt_2$ and $rt_3$. We subtracted $Wasserstein(rt_2, rt_3)_{test}$ from $Wasserstein(rt_2, rt_3)_{baseline}$ to calculate $\Delta W_{chunk}$, the change of reaction time homogeneity.

To control for the effect of training resulting in an overall increase of reaction time homogeneity, we compared this change of Wasserstein with size 3 sub-sequences that are not the frequent size-3 chunks (ABC, BCD, CDA, DAB), as a control, to arrive at $\Delta W_{control}$ as the difference between $W_{test}$ and $W_{train}$.

Finally, we subtracted $\Delta W_{control}$ from $\Delta W_{chunk}$, to arrive at the resulting measure of “chunkiness”. This is the relative change of the Wasserstein distance of size 3 chunks $\Delta W_{chunk}$ compared to control $\Delta W_{control}$. According to the model prediction, if participants in the fast group learned more size three chunks than those in the accurate group, one would expect the fast group to have a higher measure of chunkiness than those in the accurate group. Figure 4c shows the resulting chunkiness measure. The change $\Delta W$ on size 3 chunks differed significantly between the two groups ($\chi ^2(1) = 4.71$, $p = .02$), with the fast group showing a higher chunkiness compared to the accurate group ($\hat{\beta } = 12.33$, $t(88) = 2.15$, $p = .03$). Thus, participants in the fast condition showed a higher relative chunkiness in their reaction times to size three chunks in the sequence than participants in the accurate group, as indicated by the chunkiness measure.

### Reaction time regression

Our model simulations showed that when the speed-accuracy trade-off parameter $w \rightarrow 0$ and accuracy becomes the only optimizing term, the model learns about the original transition matrix. However, as $w \rightarrow 1$ and speed is the only optimization term, the model learns a polarized transition probability where all the high and medium single element transitions become 1.

We aimed to test whether or not the $w$ parameter of our model captured how the “fast” versus “accurate” instructions affected participants’ chunking behavior. In this way, the instruction will influence how participants perceive the items in the test block, the polarized transition should resemble more of the fast group, and the original transition matrix shall resemble more of the reaction time in the accurate group. An illustration of this procedure is shown in Fig. 6 in SI.

To this end, we fitted a linear mixed-effects regression to participants’ log reaction times in the correct trials of the test blocks (so that the skew of RT distribution as deviating from a normal distribution is removed), assuming a random intercept for each participant. The independent variables are the group, the true transition probabilities learned with $w = 0$ (Fig. 2d left), and chunky transition probabilities that correspond to the learning result of the model with $w = 1$ (Fig. 2d right). The best regression model contained the main effects of the chunky and true transition probabilities as well as two interaction effects with the given condition ($\chi ^2(3) = 34.86$, $p < 0.001$). The first interaction was between the true probabilities and group (see Fig. 4d; $\hat{\beta } = -0.03$, $t(16740) = -2.74$, $p = 0.006$), as the true transition probabilities were more consistent with participants’ responses in the accurate group than with those in the fast group. The second interaction was between the chunky transition probabilities and group ($\hat{\beta } = 0.05$, $t(16740) = 4.13$, $p < .001$), indicating that the simulated effect of a higher tendency to chunk was more predictive of the behavior of the fast group than that of the accurate group. Thus, the chunking bias induced by the speed-accuracy trade-off parameter of our model matched the bias observed in the reaction time pattern of the participants under speed demands.

### Chunk increase

The rational model of chunking that trades off speed with accuracy learns longer chunks as the emphasis on speed weights more than accuracy. It predicts that the fast group should learn longer chunks more often than the accurate group. This acquisition of longer chunks during the 6 training blocks should influence participants’ chunking behavior in the test blocks relative to the baseline blocks. A concrete examination of this prediction is to take the frequency of concrete size 2 and size 3 chunks in the test block to see if they are used more often compared to the baseline blocks.

Similar to experiment 1, the exact chunks learned by participants are tagged using the mixture of Gaussian method. We studied the number of times size 2 chunks appear in participants’ chunking profiles (AB, BC, CD, DA) and size 3 chunks (chunk ABC, BCD, CDA, DAB). We compared the increase in those chunks from the baseline and the test blocks and compared ﻿$\Delta N$ across the two groups. Shown in Fig. 4e is the increase in the number of size 2 and size 3 chunks.

Fitting a linear model on $\Delta N$ with group as the independent variable revealed a significant effect of group ($F(1) = 8.13$, $p = 0.005$). Compared to the accurate group, the fast group acquired more chunks from the baseline to the test block ($\hat{\beta } = 6.41$, $t(358) = 2.85$, $p = 0.005$). Consistent with the model prediction that the fast group should chunk longer chunks, participants in the fast group indeed has a higher increase in the number of size 2 and size 3 chunks compared to the accurate group.

### Chunk reuse

We also look at whether participants tend to reuse previously learned chunks to construct new chunks during the training blocks, similar to what we tried in experiment 1, to see whether the participant’s behavior reflects the feature of this model. The progress of chunk learning as identified by the mixture of Gaussian method is used to examine participants’ chunk reuse probability (illustrated in Fig. 8 in SI). The chunk reuse probability was evaluated on chunks of size three or bigger (excluding chunks with single-item repetitions). Every time such a chunk occurs in the sequence, we check whether it reuses any of the 30 previous chunks. By tagging each chunk learned by the participant as reusing one of the previous chunks or not, we arrived at a chunk reuse probability for each participant across the fast and accurate group. Figure 4f shows the average chunk reuse probability across the three groups. Participants’ high reuse probability echoes the model feature of reusing previously learned chunks to construct new ones. Interestingly, instruction also influences the tendency of chunk reuse. Fitting a linear model to participants’ chunk reuse probability showed that chunk reuse differed significantly between the two groups ($F(1) = 4.75$, $p = .03$). Participants in the fast group reuse chunks more frequently than those in the accurate group ($\hat{\beta } = 0.07$, $t(114) = 2.18$, $p = .03$). This may show that reuse is especially prominent when participants are trying to be fast, since recycling the previously learned chunks can make more progress towards reaction time speed-up.

## Discussion

How people perceive and extract structure from a sequence of perceptual stimuli has been a longstanding question of psychological investigations. *Chunking* has been proposed as a mechanism to identify repeated patterns and segment sequences into those patterns. This way of segregating patterns into discrete chunks can improve storage, retrieval, and planning across multiple psychological domains.

In the current work, we have proposed that chunking benefits the timely and accurate execution of sequential actions. We used a rational model of chunking that adapts its representation to optimize a trade-off between speed and accuracy to simulate chunk learning in a serial reaction time task. Our simulations predicted that participants should chunk more if chunks are indeed part of the generative model and should, on average, learn longer chunks when optimizing for speed than accuracy. We tested these predictions in two experiments. In Experiment 1, participants learned from sequences with different embedded chunks. In Experiment 2, participants were instructed to act as fast or accurately as possible. Multiple measures of chunking confirmed our model’s predictions in both experiments. In summary, our results shed new light on the benefits of chunking and pave the way for future studies on step-wise representation learning in structured domains.

The model’s prediction relating chunking to reaction time speed up relied partially on the Linear Ballistic Accumulator framework to translate within-chunk action prediction to an elevated starting point of the evidence accumulation, making the within-chunk action more likely to cross the decision threshold. Yet it remains challenging to explicitly fit a hierarchical LBA model over all participants, trials, and between-subject differences using our current data. This divergence is potentially due to a large number of observations. Therefore, one part of our analyses used model-predicted transition probabilities with accuracy and speed extremes to fit participants’ reaction times. Nonetheless, future studies should look into the influence of chunking on the starting point of the LBA model in a fully Bayesian and hierarchically-structured model.

Currently, our model’s predictions were primarily qualitative, and we did not compare across a more extensive set of alternative models. Even though we tested model-specific predictions such as the reuse of previously created chunks to parse the sequence and the speed-up and increased homogeneity of reaction times for within-chunk reaction times, future studies should further compare explicit predictions of different chunking models. We believe that our current work is a concrete first step towards building fine-grained models of human chunking in SRTs. We plan to compare our model to several alternatives in future tasks requiring participants to learn increasingly more hierarchically-structured chunks.

Furthermore, it would be very hard to exclude the contribution of associative learning to the effect observed in Experiment 1, as the rational chunk learning model also learns chunks by association. However, an associative learning model does not explain our observation in Experiment 2, which can only be accounted for by a rational chunking model that trades off speed with accuracy.

Finally, not all of our measures of increased chunking provided evidence for our model’s predictions. In particular, in Experiment 1, the measure of chunkiness did not increase for the size 3 group even though we would have a priori expected such an increase. We believe that this increase did not appear because participants’ speed-up of within-chunk reaction times was not uniform across both transitions of the size three chunks. Moreover, we did find a decrease of homogeneity for the size 2 group, which was as expected because learning about the size 2 chunk should make the reaction time discrepancy between B and C larger. Importantly, we did find systematic differences across all other measures in both experiments and, therefore, believe that the current data support our model’s predictions.

## Conclusion

We investigated chunking behavior across two experiments and several measures. We found that chunking behavior depends on sequence statistics and task demands. When there are chunks in the training sequence, participants learn the underlying embedded chunks. Additionally, task demands modulate chunking behavior. Participants tend to chunk more when they are optimizing for speed rather than accuracy. Such chunking behavior occurs even in sequences lacking any deterministic transition probabilities. Our results suggest characteristics of chunking and how they interact with task demands. Our rational model of chunking captures and predicts these findings. The success of model predictions depends primarily on the gradual change in previously acquired representations to rationally adapt to sequence structure and task demands. We hope that our findings and model are a good step towards understanding human chunk learning across multiple domains.

## Methods

### Ethics statement

Informed consent was obtained from all participants before participation, and the experiments were performed in accordance with the relevant guidelines and regulations approved by the ethic committee of the University of Tuebingen (Ethik-Kommission an der Medizinischen Fakultät der Eberhard-Karls-Universität und am Universitätsklinikum Tübingen), under the study title: Experimente zum Sequenz- und Belohnungslernen, with application number 701/2020BO.

Participants’ data were analyzed anonymously. Upon agreement to participate in the study, they consented on a data protection sheet approved by the data protection officer of the MPG (Datenschutzbeauftragte der MPG, Max-Planck-Gesellschaft zur Förderung der Wissenschaften).

### Recruitment of participants

For Experiment 1, we recruited 142 participants from Amazon Mechanical Turk, out of which sixty-nine were female. Their median age was between 30 and 40, and the overall age ranged from 18 to above 50. This experiment took around 25 minutes to complete. After completing the task, participants received a base pay of $2 and a performance-dependent bonus of up to $6.

For Experiment 2, we recruited a total of 116 participants for our study, again from Amazon Mechanical Turk. Forty-eight participants were female; participants’ median age was between 30 and 40, and the overall age ranged from 18 to above 50. After completing the task, participants received a base pay of $2 and a performance-dependent bonus of up to $4.

### Payment

For Experiment 1, a performance-dependent bonus was calculated as the weighted sum of participants’ accuracy and reaction times. When the average accuracy was below 70%, the bonus was set to 0. The bonus for being fast was calculated as $$bonusfast = bonusmax - (\overline{rt} - 600) \times 0.025$$, where $$\overline{rt}$$ indicates the average reaction time and *bonusmax* indicates the maximal bonus. Participants were rewarded with a reaction time bonus when their average reaction time was below 600ms. Additionally, an accuracy bonus was calculated as the mean performance accuracy times the maximal bonus, $$bonusacc = \overline{acc} \times bonusmax$$. At the end of the experiment, the total bonus was calculated as a weighted average between the bonus for participants’ accuracy and the bonus for their reaction times, $$bonus = 0.5 \times bonusacc + 0.5 \times bonusfast$$. If the final bonus was below 0, it was set to 0. If it was above the maximal bonus, it was set to the maximal bonus. On average, participants received $5.64 for their participation.

For Experiment 2, USD 2 were awarded as a base pay for every participant who completed the experiment. Additionally, participants received a performance-dependent bonus, ranging from 0 to the maximum of USD 4. This bonus was calculated separately for the fast and accurate groups. For the fast group, the bonus was 0 when their average accuracy was below 60%. If the average accuracy was above 60%, then the bonus was calculated as $$bonusfast = (1000 - \overline{rt})/800 \times bonusmax$$ This reward function penalized average reaction times that were slower than 1000ms. For the accurate group, the bonus was calculated as the percentage of their accuracy multiplied by the maximal bonus for the accuracy group, $$bonusacc = \overline{acc} \times bonusmax$$. Finally, the final bonus was again forcet to be between 0 and *bonusmax* (USD 4). The mean reward earned by participants for this experiment was $4.16.

### Filtering criteria

We decided to discard participants using a fixed RT threshold based on independent pilot data we had collected earlier. Since the study was conducted on MTurk, and we do not have access to the conditions on how the experiment was conducted, we applied stricter filtering criteria. For Experiment 1, we excluded participants with an average reaction time longer than 1000ms or an average accuracy lower than 90%. 95.1% of participants had an accuracy above 90%. 91% of participants had an average reaction time below 1000 ms. Out of 142 participants who participated in Experiment 1, 20 participants were excluded and 122 remained after applying this exclusion criterion.

For Experiment 2, the same exclusion criteria were applied on the baseline and test blocks when both groups were asked to be as fast and accurate as possible. 96.7% of participants had an accuracy above 90%, and 96.7% had an average reaction time below 1000 ms. The exclusion criteria differed between the two groups on the training blocks. Participants in the fast group were excluded when the average reaction time was above 750ms ($n=13$). Those in the accurate group were excluded when their accuracy was below 90% ($n=10$). Additionally, we excluded participants who repeatedly failed attention checks before and after the experiment ($n=3$). Out of 116 participants, 26 were excluded in total. All of the following analyses were performed on the data of the remaining 90 participants.

For the reaction-time based analysis including the chunky boost, chunkiness, and the mixture of Gaussians classification, we further excluded trials in which participants took more than 1000ms to respond. This amounted to $8.4\%$ of all trials in Experiment 1, and $3.3\%$ of all trials in Experiment 2.

### Mixture of Gaussians model

We used a mixture of Gaussians model to retrieve chunky transitions for each participant’s responses from their reaction times. Chunks are classified based on participants’ responses, irrespective of their correctness. In the case of an error where a participant has pressed A B D C although the instruction was A B D B, and the reaction time classification for each of these trials are between, within, within, within (and the subsequent trial is between again), then A B D C is classified as a chunk. In the case of errors, we consider their erroneous response rather than the instruction because the response reflect their underlying prediction.

The reaction time distribution for individual participants was used to classify individual trials as between or within-chunk reaction times. These reaction time distributions were fitted by a mixture of Gaussians model. The likelihood of belonging to the smallest mixture component was used to classify a reaction time as a within-chunk reaction time. This classification was then used for the identification of chunks for all experimental trials of every participant.

The classification of RTs by using multi-modal distributions was motivated by the idea that distinct processes might generate the within-chunk and between-chunk reaction times. During an SRT trial, if the participant has no expectation for the next upcoming instruction, she will first have to identify the instructed key on the screen before beginning to press a key. This will make her between-chunk reaction times larger. In contrast, if a participant has learned chunks, she anticipates the next instruction before it is even shown. If the upcoming instruction is within her expected chunk, the action to look for instructions displayed on the screen can be omitted, and she can directly engage in pressing their expected subsequent key. This will make her within-chunk reaction times smaller. Additionally, the mixture of Gaussians model also takes into account participants’ post-error slow-downs. These correspond to the trials when a participant has made or almost made a mistake and corrects this tendency to press an expected key upon the observation of a conflicting instruction. The behavior of modifying the wrong key-press is slowing down the reaction time even more.

Because these three processes contribute to distinct components of participants’ reaction times, a mixture of 3 Gaussian distributions was used to fit their reaction time distributions. We assumed that the within-chunk reaction time distributions had the lowest mean, the between-chunk reaction time distributions had a higher mean, and the post-error slow-down reaction time distribution had the highest mean. We fitted the mixture of Gaussian model to individual participants’ reaction times, filtering out RTs above 1000ms. A likelihood estimate belonging to each distribution amongst the mixture was assigned to the reaction times of each trial. A validation of this method has been included to the Supplementary Information.

## Supplementary Information

Supplementary Information.

## Data Availability

The data collected and code used for analyzing this study can be found in this github repository: https://github.com/swu32/experimental_chunking.
