# Supplementary material for: Chunking as a rational solution to the speed–accuracy trade-off in a serial reaction time task
Source: Sci Rep. 2023 May 11;13:7680. doi: 10.1038/s41598-023-31500-3 (PMC10175304; doi:10.1038/s41598-023-31500-3)
Supplement: Supplementary file 1 — Supplementary Information. [file 41598_2023_31500_MOESM1_ESM.pdf]

Supporting Information: Chunking as a rational solution to the speed-accuracy trade-off in a serial reaction time task

Shuchen Wu[1,*], Noémi Éltető[2], Ishita Dasgupta[3], and Eric Schulz[1]

[1]MPRG Computational Principles of Intelligence, Max Planck Institute for Biological Cybernetics, Tübingen, Germany
[2]Department of Computational Neuroscience, Max Planck Institute for Biological Cybernetics, Tübingen, Germany
[3]Google DeepMind, New York City, New York, USA
[*]shuchen.wu@tue.mpg.de

Calculation of Chunky Boost

Figure 1 illustrates how Chunky Boost is calculated from reaction time data. As an example, when the relevant chunk is AB, we took the rt of pressing B (within-chunk press). The Cohen's d was measured on the speed-up between the baseline and the test block of such within chunk key presses:

$$d_{AB} = \frac{\bar{rt}_{baseline} - \bar{rt}_{test}}{\sigma} \tag{1}$$

σ was the standard deviation of rt.

The control reaction times came from size 2 subsequences that did not start with A, and the reaction times were measured on the second item of the subsequence, as illustrated in Figure 1. Cohen's d was again evaluated as:

$$d_{AB_{control}} = \frac{\bar{rt}_{baseline} - \bar{rt}_{test}}{\sigma} \tag{2}$$

The chunky boost was calculated by subtracting the control from the Cohen's d measured on relevant chunks:

$$\Delta d = d_{AB} - d_{AB_{control}} \tag{3}$$

For Experiment 2, the relevant chunks were AB, BC, CD, and DA, the control subsequences were all size 2 subsequences excluding the relevant chunks.

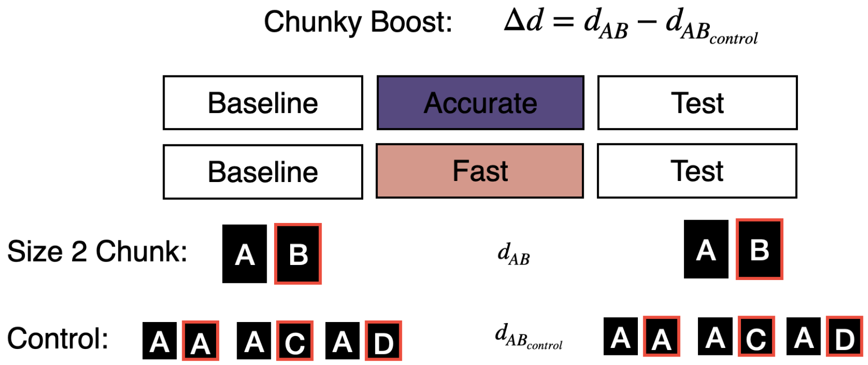

Figure 1. An illustration of the measurement of chunky boost.

Calculation of Chunkiness

When the relevant size 3 chunk was ABC, the Wasserstein distance was evaluated on rt_B and rt_C separately as $Wasserstein(rt_B, rt_C)$. The chunkiness measure then was

$$\Delta W_{ABC} = Wasserstein(rt_B, rt_C)_{baseline} - Wasserstein(rt_B, rt_C)_{test} \tag{4}$$

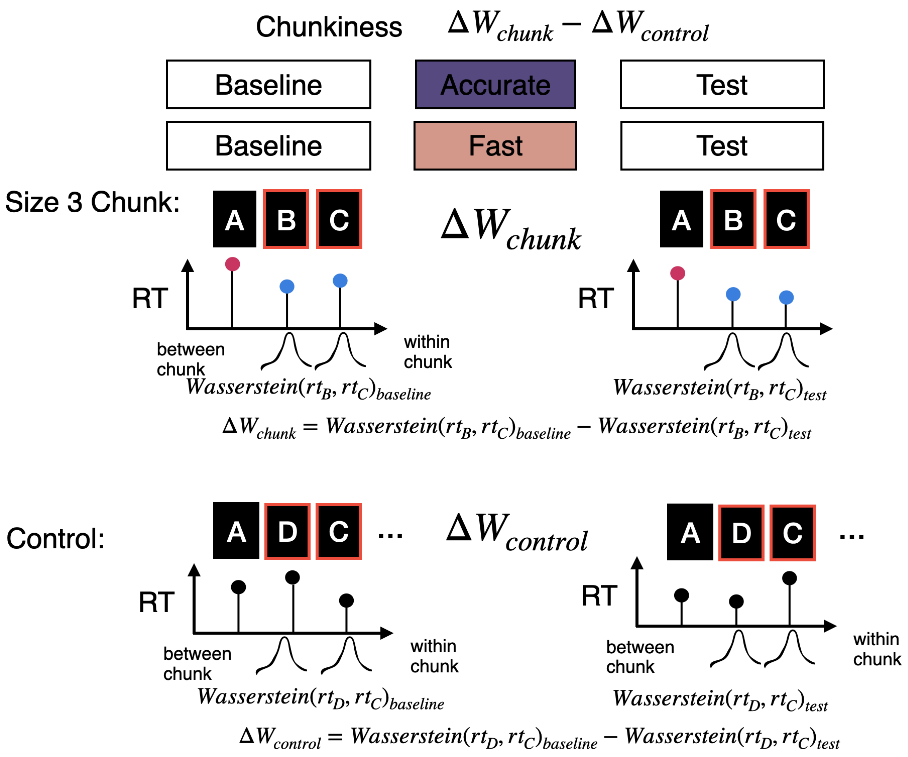

Figure 2. An illustration of the measurement of chunkiness.

The control chunks were subsequences that were not ABC, and their chunkiness measure became:

$$\Delta W_{control} = Wasserstein(rt_2, rt_3)_{baseline} - Wasserstein(rt_2, rt_3)_{test} \tag{5}$$

rt_2 and rt_3 denote the second and the third reaction time of the size 3 subsequences used as control. The measure of chunkiness is the control subtracted from the relevant chunks.

$$Chunkiness = \Delta W_{ABC} - \Delta W_{control} \tag{6}$$

For Experiment 2, the relevant chunks were ABC, BCD, CDA, DAB, and the controls were all size 3 subsequences that were not any of the chunks. Chunkiness was evaluated in the same way.

Mixture of Gaussians Model

The mixture of Gaussians method categorizes the reaction time profile of each participant into within- or between-chunk reaction time as illustrated in Figure 3a. For each participant, the reaction time distribution was fit via a mixture of three Gaussians. When the likelihood of belonging to the mixture component with the lowest mean exceeds that of the other two components, the reaction time is classified as within-chunk.

When fitting the mixture of Gaussians model to individual participants' data, we separately varied the number of mixtures from 1 to 3 and used the Akaike Information Criteria (AIC) and Bayesian Information Criteria (BIC) to evaluate the three models. Shown in Figure 3b is the distribution of the best fitting number of mixtures over all participants across the two experiments. For most participants and both evaluation criteria, the reaction time distribution was best described using 3 mixture components.

Validation via Simulated RT Distributions

To perform a validation study on this method, we simulated within- and between-chunk reaction time distributions by generating two exponentially modified Gaussian distribution (ex-Gaussian) with distinct mean and standard deviations. The exponentially modified Gaussian distribution normally provides a good fit for the reaction time distributions, as the simulated RT is straightly

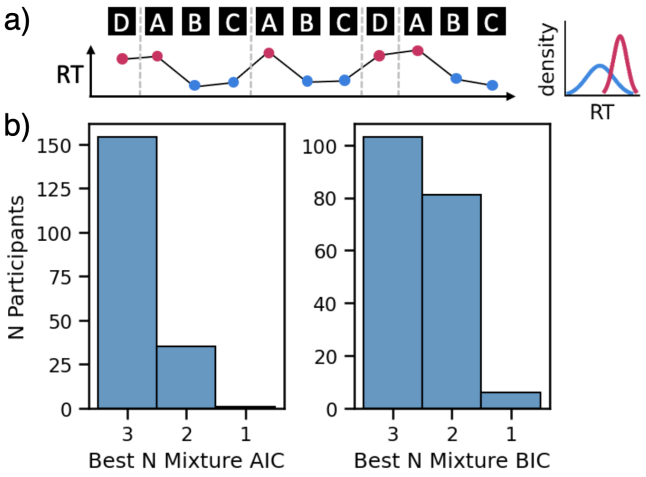

Figure 3. Mixture of Gaussians model for reaction time classification. a) Individual participant's reaction time of reacting upon key-press instructions across time. The distribution (right) is modelled as a mixture of two or three Gaussians. b) Histogram of the number of mixtures that led to best model selection criteria across all participants. Top: Best number of mixtures based on BIC (Bayesian Information Criteria). Bottom: Best number of mixtures based on AIC (Akaike Information Criteria).

positive, and the exponential decay component takes account of the observed right-skewness of RT distributions[1,2]. The ex-Gaussian distribution has the following probability density function:

$$f(x;\mu,\sigma,\lambda) = \frac{\lambda}{2} e^{\frac{\lambda}{2}(2\mu+\lambda\sigma^2-2x)} erfc\left(\frac{\mu+\lambda\sigma^2-x}{\sqrt{2}\sigma}\right) \tag{7}$$

$erfc$ is the complementary error function defined as:

$$erfc(x) = \frac{2}{\sqrt{\pi}} \int_x^{\infty} e^{-t^2} dt \tag{8}$$

μ is the mean of the independent Gaussian variable, σ is the standard deviation, and τ is the mean of the exponential component. The first three moments of the resulting ex-Gaussian distribution are $\mu+\tau$, $\sigma^2+\tau^2$ and $2\tau^3$.

We fixed the dispersion parameter (λ) to be 1. We varied the difference in the mean μ between the within and between-chunk distributions, in addition to the spread parameter σ. After randomly interspersing samples coming from the two distributions across 1000 trials (the same length as in the experiments), we used the mixture of three Gaussians to classify the simulated rt data. Plotted in Figure 4 is the classification accuracy with increasing mean differences between the two simulated rt distributions varying spread parameters σ (σ_w for within-chunk and σ_b for between-chunk distribution). Note that for most of the parameters, the worst classification accuracy is above 75%.

Chunk Growth Rate Experiment 1

After obtaining the chunking profile of every participant as reflected in their reaction time speed-ups (as demonstrated in Figure 5), we evaluated the growth rate of chunks in the three groups of participants by looking at how fast chunk size increases (Figure 7). Figure 9 shows the rate of chunk increase between the three groups. Fitting a linear mixed-effects regression onto participants' chunk size, assuming random intercepts for each participant, showed a significant effect of trial number ($\chi^2(1) = 51.22, p < 0.001$). Generally, the rate of increase was positive, suggesting that the chunk size acquired by participants grew as a function of time. The chunk increase rate also differed significantly between the three conditions. The size 2 group ($\hat{\beta} = 1.43\times10^{-4}, t(84700) = 2.38, p = 0.02$) and the size 3 group had a significantly higher rate of chunk size increase compared to the independent group ($\hat{\beta} = 1.36\times10^{-4}, t(84700) = 2.93, p = 0.003$). To summarize, the chunk size acquired by participants increased over time and differed across the three groups. This observation is consistent with the design of the rational chunking model which assumes that participants reuse the previously learned chunks to construct longer chunks during the task.

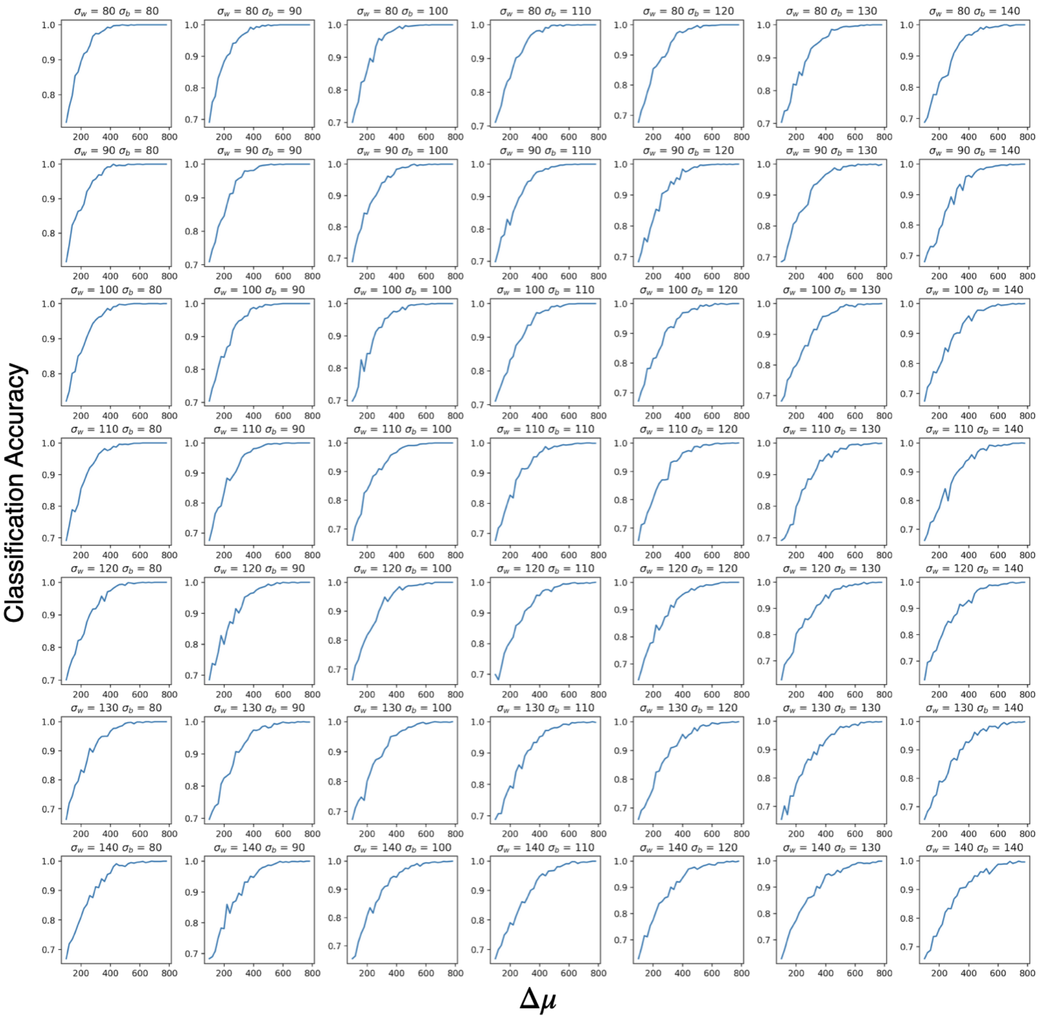

Figure 4. Classification accuracy (y-axis) of the mixture of Gaussians model on simulated reaction time data with increasing difference in mean $\Delta\mu$ (x-axis) and varying the standard deviation of the within-chunk (σ_w) and between-chunk (σ_b) distributions. σ_w increases from the top to the bottom, and σ_b increases from the left to the right.

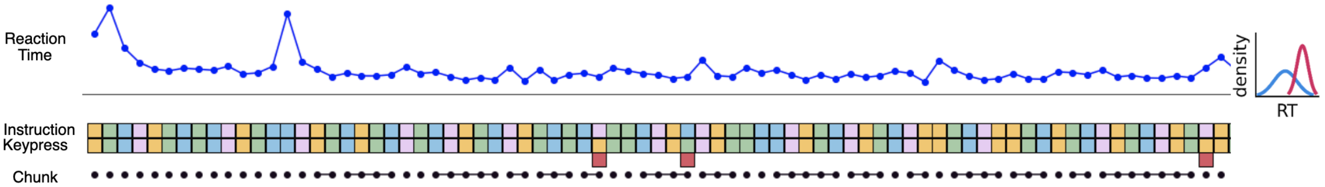

Figure 5. Chunk learning profile of one participant. The first, second, and third row show reaction times, instruction displayed, and this participant's key press (A, B, C, D, are separately color-coded by green, blue, magenta and orange boxes). We look at the exact chunks used by participants by classifying within and between chunk reaction time using the mixture of Gaussian method (see section method). When the participant pressed an incorrect key, that trial is marked by a red box in the fourth row. Using the distribution of reaction time data accumulated for this participants over all trials, we classify individual trials into within or between-chunk key presses, based on the likelihood assigned by the mixture model. Chunks learned by this participant are marked by connecting each between-chunk trial with the subsequent within-chunk trials, displayed by connected black dots in the last row.

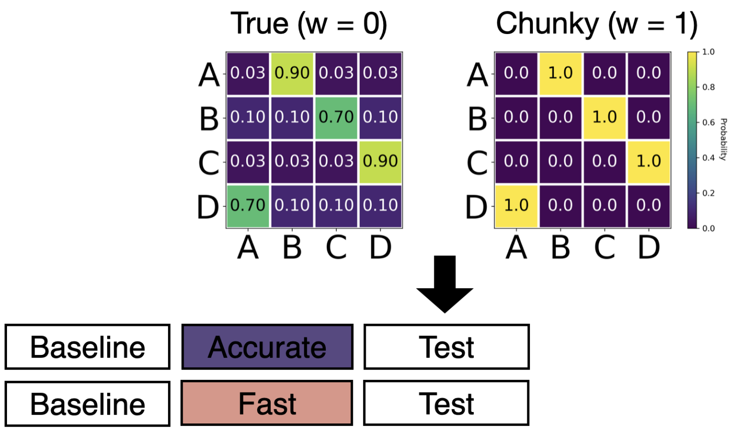

Figure 6. Transition probability regression.

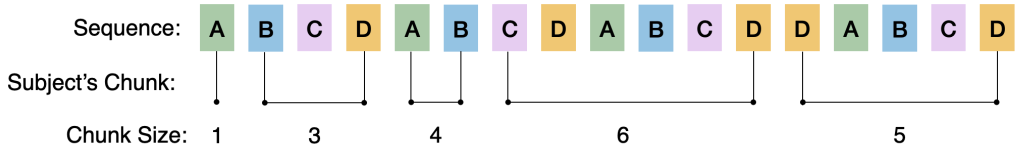

Figure 7. Measurement of chunk size.

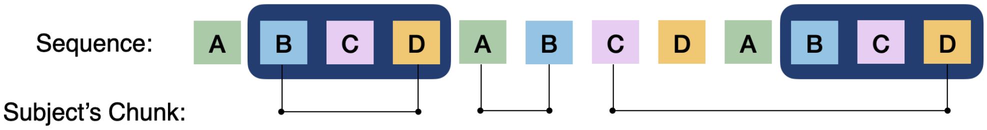

Figure 8. Chunk reuse measurement. After the classification of chunks, we look back in history to evaluate the probability of each chunk reusing the components of the previous chunks. In this case, chunk CDABCD is reusing the previous chunk BCD.

Chunk Growth Rate Experiment 2

We also evaluated the rate of increase of every participant in experiment 2, as illustrated in Figure 9. Fitting a linear mixed-effects regression onto participants' chunk size assuming random intercepts of participants also showed a significant effect on trial number ($\chi^2(1) = 778.82, p < 0.001$). Additionally, there was an interaction effect between the groups and trial numbers. The fast group had a higher chunk learning rate than the accurate group ($\hat{\beta} = 2.68 \times 10^{-4}, t(62870) = 4.887, p < 0.001$). This result suggests that the fast group had a higher tendency to build chunks than the accurate group, which is consistent with model prediction.

Acknowledgements

We thank Peter Dayan, Felix Wichmann, and Mirko Thalmann for helpful discussions. This work was supported by the Max Planck Society.

Code and Data Availability Statement

The data collected and code used for analyzing this study can be found in this github repository: `https://github.com/swu32/experimental_chunking`

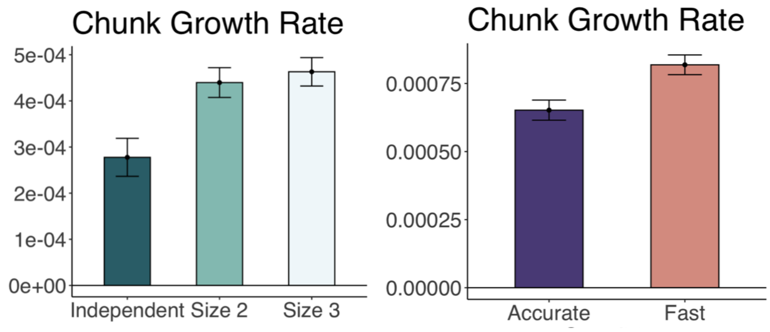

Figure 9. Average chunk growth rate for Experiment 1 (left) and Experiment 2 (right)

Additional Information

The authors have declared that there are no competing interests.

References

1. Heathcote, A. Rtsys: A dos application for the analysis of reaction time data. *Behav. Res. Methods, Instruments, & Comput.* **28(3)**, 427–445, DOI: 10.3758/BF03200523 (1996).

2. Burbeck, S. L. & Luce, R. D. Evidence from auditory simple reaction times for both change and level detectors. *Percept. & Psychophys.* **32(2)**, 117–133, DOI: 10.3758/BF03204271 (1982).

Author Contributions

Conceptualization: Shuchen Wu, Noémi Éltető, Ishita Dasgupta, Eric Schulz.
Formal analysis: Shuchen Wu, Eric Schulz.
Software: Shuchen Wu.
Visualization: Shuchen Wu, Noémi Éltető.
Writing – original draft: Shuchen Wu, Eric Schulz.
Writing – review & editing: Shuchen Wu, Noémi Éltető, Ishita Dasgupta, Eric Schulz.
